# Unraveling the complexity of STAT3 in cancer: molecular understanding and drug discovery

**DOI:** 10.1186/s13046-024-02949-5

**Published:** 2024-01-20

**Authors:** Yamei Hu, Zigang Dong, Kangdong Liu

**Affiliations:** 1 Tianjian Laboratory for Advanced Biomedical Sciences, School of Basic Medical Sciences, Zhengzhou University, Zhengzhou, 450001 Henan China; 2 Medical Research Center, The Third Affiliated Hospital of Zhengzhou University, Zhengzhou University, Zhengzhou, 450052 Henan China; 3 China-US (Henan) Hormel Cancer Institute, Zhengzhou, 450008 Henan China; 4 State Key Laboratory of Esophageal Cancer Prevention and Treatment, Zhengzhou, Henan China; 5 Provincial Cooperative Innovation Center for Cancer Chemoprevention, Zhengzhou, Henan China; 6 Cancer Chemoprevention International Collaboration Laboratory, Zhengzhou, Henan China

**Keywords:** STAT3, Cancer, Modulators, Metabolic reprogramming, Immunosuppression, Inhibitors

## Abstract

Signal transducer and activator of transcription 3 (STAT3) is a transcriptional factor involved in almost all cancer hallmark features including tumor proliferation, metastasis, angiogenesis, immunosuppression, tumor inflammation, metabolism reprogramming, drug resistance, cancer stemness. Therefore, STAT3 has become a promising therapeutic target in a wide range of cancers. This review focuses on the up-to-date knowledge of STAT3 signaling in cancer. We summarize both the positive and negative modulators of STAT3 together with the cancer hallmarks involving activities regulated by STAT3 and highlight its extremely sophisticated regulation on immunosuppression in tumor microenvironment and metabolic reprogramming. Direct and indirect inhibitors of STAT3 in preclinical and clinical studies also have been summarized and discussed. Additionally, we highlight and propose new strategies of targeting STAT3 and STAT3-based combinations with established chemotherapy, targeted therapy, immunotherapy and combination therapy. These efforts may provide new perspectives for STAT3-based target therapy in cancer.

**Supplementary Information:**

The online version contains supplementary material available at 10.1186/s13046-024-02949-5.

## Introduction

STAT3 belongs to the STATs family and comprises STAT1, STAT2, STAT3, STAT4, STAT5a, STAT5b and STAT6 that share similar structures and functional domains [1]. STAT3 is one of the most well-described STATs members and which mainly acts as tumor promoting roles in tumor development and progression. Apart from the canonical functions such as proliferation, apoptosis, metastasis, angiogenesis, drug resistance, self-renewal of cancer stemness, the newly identified functions such as epigenetic regulation, immune surveillance, tumor inflammation, metabolic reprogramming and exosome-related biological activities also contribute to the oncogenic roles in cancer.

STAT3 historically has been considered “undruggable”. However, the current development of advanced technologies and novel therapeutic strategies in STAT3 inhibitors has moved toward “druggable”. A series of selective inhibitors that directly or indirectly target STAT3 have been identified in the past three decades. Excitingly, most of these inhibitors show excellent tumor inhibitory effects in preclinical and clinical trials. However, no clinically applicable drugs that directly targeting STAT3 has been approved for clinical use so far. In this review, we focus on the new progress in studies of regulation of STAT3 and its essential roles in various biological regulations. We also provide a summary of the selective inhibitors tested in various preclinical and clinical studies for cancer treatment.

## Overall reviews of STAT3

### Isoforms of STAT3

**Fig. 1:**
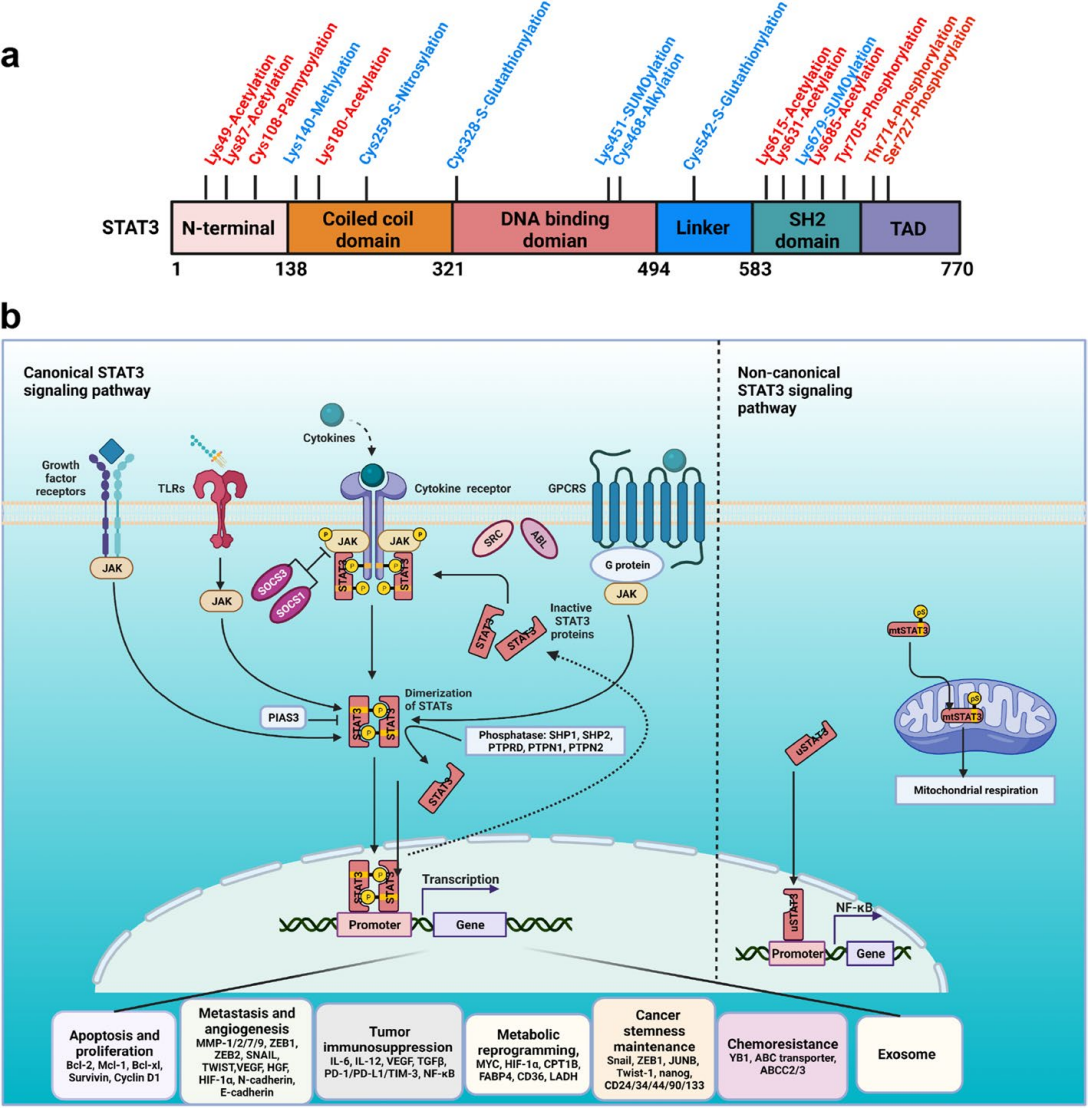
STAT3 structure and the canonical and non-canonical STAT3 signaling pathways in cancer. **A** Diagrams of structure and function domains of STAT3 with the posttranslational modification residue sites. STAT3 is composed of an N-terminal domain (NTD), DNA binding domain, linker domain, Src homology 2 domain (SH2), and a C-terminal transactivation domain (TAD) with a tyrosine phosphorylation residue at 705 and a serine phosphorylation residue at 727. Red font represents activation PTM sites, blue font represents inactive PTM sites. **B** Left part, the canonical STAT3 signaling pathway is activated by multiple receptors including interleukin-6 (IL-6) and IL-6 family cytokines (IL-11, IL-23) receptors, G-coupled receptors (GPCRs), growth factor receptors and Toll-liker receptors which are stimulated by cognate ligands varies from cytokines, hormones, angiotensin, sphingosine-1-phosphate, and LPS et al. Traditionally, these receptors lack the intrinsic kinase activity. Once the ligands recognize the cognate receptors, the ligand-receptor shifts conformation and activate the JAKs, then provide the anchor site for STAT3 to bind via its SH2 domain. In addition to being activated by the membrane receptors, the constitutive activation of STAT3 is also induced by oncoproteins with tyrosine kinase activity like SRC and BCR-ABL. Two phosphorylated STAT3 form dimers via the phosphorylated tyrosine residue 705 of one monomer interacts with the SH2 domain of another monomer, the dimers subsequently translocate into the nucleus and bind to the specific DNA response elements in the promotor regions of the target genes which are involved in proliferation, metastasis, angiogenesis, tumor immune suppression, metabolic reprogramming, cancer stemness, drug resistance and exosome activity. Right part, the non-canonical STAT3 signaling pathway has three forms including mtSTAT3, unphosphorylated STAT3 and *p*-STAT3 Ser727 either alone or together with *p*-STAT3 Tyr705. These forms of STAT3 regulate the mitochondrial respiration, NF-κB, and other unknown genes are involved in activities. Created with BioRender.com

Structurally, STAT3 mainly consists of the N-terminal domain (NTD), coiled-coiled domain, DNA binding domain (DBD), linker domain, Src homology 2 (SH2) domain and transactivation domain (TAD) [2]. The tyrosine residues 705 and serine residues 727 located in the C-terminal are considered two primary function activation sites, as shown in Fig. 1a. STAT3 gives rise to six isoforms, STAT3α, STAT3β, STAT3γ, STAT3δ, STAT3ε and STAT3ζ (shown in Table 1). These isoforms determine the distinct functions of STAT3. STAT3α is the longest isoform, commonly designated as STAT3, and contributes most to the canonical functions of STAT3. STAT3 consists of 24 exons. STAT3β is generated by alternative splicing at exon 23, causing a frameshift and the transactivation domain is replaced with seven specific amino acids [8]. Conversely, STAT3β plays a tumor suppressive role due to the lack of transactivation domain and commonly predicts a favorable outcome in tumor patients [9]. A recent review of the literature reported that STAT3β cooperates with STAT3α and co-activators to form a ternary complex called “spongy cushion”. When the STAT3β keeps a relatively high-level in the ternary complex, STAT3β suppresses proliferation and self-renewal, attenuates invasion, lessens chemotherapy resistance and induces apoptosis in cancer [10]. The STAT3γ and STAT3δ isoforms are derived from the proteolytic processes which are associated with the maturation of neutrophil and granulocyte in different stages [3–5]. STAT3ε and STAT3ζ, are two novel putative truncated forms of K685-acetylated STAT3α. STAT3ζ is reported to promote cardiomyocyte formation via acting as an adaptor to ErbB4-p38γ signaling cascade. Additionally, N-terminal containing STAT3ε and C-terminal containing STAT3ζ share overlapping homology with STAT3α [6]. However, the detailed functions of STAT3ε need further studies. In this review, we mainly focus on the progresses in research of STAT3α.

**Table 1:** Isoforms of STAT3

STAT3 isoforms	Function	Structure	MW (KDa)	Generating process	Ref
STAT3α	Canonical activator of transcription; Key roles during STAT3 promoting cancer development	The longest isoform	92	Alternative splicing	[2]
STAT3β	Repressor of STAT3; Higher DNA-binding affinity; Higher stability of STATs dimers	Loss of C-terminal (Ser727 missing)	83	Alternative splicing	[2]
STAT3γ	Regulation of neutrophil survival	Loss of C-terminal (Tyr705 and Ser727 missing)	72	Proteolysis	[3–5]
STAT3δ	Activated during neutrophils and granulocyte maturation (early stage)	Loss of C-terminal (Tyr705 and Ser727 missing)	64	Proteolysis	[3]
STAT3ε	Played no role in cardiogenesis	N-terminal containing; Shares overlapping homology with STAT3α	50	Caspase-3 cleavage upon K685-acetylation	[6]
STAT3ζ	Adaptor of the ErbB4-p38γ signaling pathway during the cardiomyocyte differentiation	C-terminal containing; Shares overlapping homology with STAT3α	45	Caspase-3 cleavage upon K685-acetylation	[6, 7]

*MW* Molecular weight, *K685* Lysine685

### The canonical and non-canonical STAT3 signaling pathways

As shown in Fig. 1b, the phosphorylation of STAT3 on tyrosine 705 is a prominent feature of canonical STAT3 activation. The canonical STAT3 signaling pathway is activated by multiple receptors stimulated by cytokines and diverse growth factors [11, 12]. Once STAT3 is activated, two monomeric STAT3 form homodimers or heterodimers via the tyrosine residue 705 reciprocally interact with the SH2 domain and subsequently translocate into the nucleus and regulate genes expression which are involved in sustaining proliferation [13], metastasis [14], angiogenesis [15], inflammation [16], resisting apoptosis [17], immune suppression [18], tumor microenvironment [16], cancer stem maintenance [19], and reprogramming metabolism [20], drug resistance [21] and exosome mediation of cancer hallmarks activities [22].

In addition to tyrosine 705, serine 727 is another vital function site. Phosphorylation of STAT3 Ser727 is triggered by serine or threonine kinases such as CDK5, JNK1/2, GSK3α/3β and MAPKs [23, 24]. Recently, non-canonical STAT3 signaling pathways have gained much attention due to their potential roles in cancer. For example, the mitochondria STAT3 (mtSTAT3) is found to be colocalized with mitochondrial electron transport chain (ETC) components [25] and alters cell metabolism, mitochondrial respiration, reactive oxygen species (ROS) production and finally promotes carcinogenesis. These processes are dependent on the phosphorylation of STAT3 Ser727 [26]. In addition to the phosphorylated form of STAT3, the unphosphorylated STAT3 (uSTAT3), in which the Tyr705 is replaced with phenylalanine, is identified interaction with unphosphorylated NF-κB or Jun activation domain-binding protein 1 (JAB1) to mediate the transcription of NF-κB and epithelial-mesenchymal transition (EMT) related genes [27, 28].

### The hyperactivation and clinical significance of STAT3/phosphorylated STAT3 in human cancers

There is considerable literature reporting that STAT3 is constitutively activated in a dozen different cancers including solid tumors (breast cancer [29], cervical cancer [30], colon cancer [31], pancreatic adenocarcinoma [32], esophageal squamous cell carcinoma [33], non-small cell lung cancer [34], ovarian carcinoma [35], et al.) and hematologic tumors (lymphomas [36], acute myeloid leukemia [37], chronic myeloid leukemia [38], et al.). However, in normal cells, the balance of STAT3 activation and inactivation is tightly controlled by transient activation and feedback inactivation of membrane receptors [39]. Elevated expression profiles of STAT3 or *p*-STAT3 Tyr705 are commonly associated with higher clinical stage, higher tumor grade, lymph node metastasis, depth of invasion, chemoresistance and worse overall survival rate or disease-free survival rate [30, 38, 40]. Based on the crucial roles of STAT3 hyperactivation in cancer development and progression established in dozen studies over the past decades, STAT3 is undoubtedly an encouraging therapeutic target. This highlights the urgent need to design select and potent inhibitors or therapeutic strategies that target STAT3 signaling pathway.

## Upstream regulation of STAT3

### Positive regulation of STAT3

As reported, hyperactivation of STAT3 commonly exists in almost all cancer types. The underlying mechanisms of elevated expression levels of STAT3 and phosphorylated STAT3 are summarized in this part. The positive regulators are listed in Table S1.

#### Ligands interact with cognate membrane receptors in STAT3 activation

As shown in Table S1, cytokines IL-6, IL-6 families (IL-11, oncostatin M, leukaemia inhibitory factor, ciliary neurotrophic factor, IL-31), growth factors (EGF, FGF, IGF, PDGF), hormones, angiotensin, sphingosine-1-phosphate, and lipopolysaccharide (LPS) are often described with activities attributed to STAT3 activation in human cancers. Most of these ligands are secreted in platelets, macrophages, fibroblasts, keratinocytes, and tumor cells, which act in paracrine, autocrine, juxtacrine, or endocrine fashions [41]. Of note, IL-6 mediates STAT3 activation and shows unique functions in androgen-dependent prostate cancer progression to the neuroendocrine differentiation stage that is clinically called neuroendocrine prostate cancer [42, 43], so far, there are no effective therapeutic strategies. IL-6 may interact with pro-inflammation or immune cells to induce endocrine effects in an autocrine/paracrine manner, which exhibits intrinsic pro-tumorigenic actions such as cell proliferation, survival, migration, invasion, metastasis and extrinsic pro-tumorigenic actions such as modulate stromal cells to shape the microenvironment and cancer inflammation by activating STAT3 [39].

#### Membrane receptors and associated kinases in STAT3 activation

G-protein coupled receptors (GPCRs) such as angiotensin II receptor and sphingosine-1-phosphate receptor (S1PR1) are the two best-known GPCRs to activate STAT3 [44, 45]. In addition, the activation of STAT3 mediated by GPCRs necessitates the involvement of JAKs. Toll-like receptors (TLRs) such as TLR2, TLR3, TLR4, TLR7, TLR9 are expressed on various immune cells, epithelial and stromal compartments and function in STAT3 mediated cancer development and progression [46]. Elevated expression of TLR2 and TLR9 are correlated with tumor grade and poor survival rates in gastric cancer patients [47] and glioma patients [48], separately. Increasing TLR3 signaling contributes to STAT3-induced upregulation of Wnt5a gene expression as well as the growth and motility of the papillary thyroid cancer cells [49]. TLR9 agonist such as CpG activates STAT3, which restrains the CpG’s immunostimulatory effects. Targeting STAT3 can improve the efficacy of TLR9 agonist-based immunotherapy, being a checkpoint or the ‘brake’ for anti-tumor immune responses [50]. Receptor tyrosine kinases (RTKs) such as EGFR, HER2/ErbB2, MET, InsR, PDGFR, VEGFR, FGFR, EphA/B, LMR and ALK play extremely important roles in human cancers [51]. And RTKs-JAKs-STAT3 signaling are well-elucidated signaling networks in cancers involving almost all cancer hallmark features [52]. In contrast to RTKs, non-receptor kinases (nRTKs) lack receptor-like properties. Besides the JAKs family (JAK1, JAK2, JAK3, TYK2) [51], the SRC family is the largest subfamily of nRTKs. The SRC activity and constitutive activating STAT3 are involved in epithelial-to-mesenchymal transition [53] and angiogenesis [54]. In addition, the oncoprotein BCR-ABL (Breakpoint-cluster region and Abelson leukemia proteins) derived from nRTKs mutation (chromosomal rearrangement) are associated with the development of hematological malignancies, either leukemia, lymphoma, or myeloma via STAT3 signaling [55, 56]. Furthermore, many serine or threonine kinases such as MAPKs, GSK3α/3β, JNK1/2, Pim-3 and ILK are reported to be responsible for phosphorylating STAT3 at serine 727 or tyrosine 705 [24, 57].

#### Long non-coding RNAs, microRNAs and circular RNAs in STAT3 activation

Increasing evidence from recent studies demonstrate that lncRNAs such as MIAT, DANCR, FLANC, lncRNA ITIH4 antisense RNA 1 (ITHI4-AS1), TNK2-AS1, PVT1 et al. promote tumor development and progression via directly or indirectly regulating STAT3 signaling [58–61]. MicroRNAs such as miR-4449, miR-182-5p, miR-221-3p, miR-203 et al. indirectly activate STAT3 mainly by negatively regulating PIAS, SOCS family members [62–65]. CircRNA is a new category of functional RNAs. Mechanistically, circRNA is identified as the miRNA sponges and suppresses the miRNAs, or interacts with RNA-binding protein (RBP) and regulates gene expression at transcriptional or post-transcriptional levels. Moreover, the circRNA also displays translational activity [66]. Circ-E-Cadherin encodes an oncogenic variant C-E-Cad through multiple-round open reading frame translation, which subsequently associates with the EGFR CR2 domain and activates STAT3 signaling pathway in maintaining the tumorigenicity of glioma stem cells [67]. Hsa_circ_0068871 targets miR-181a-5p, leading to upregulation of FGFR3 expression and ultimately promotes STAT3 signaling in bladder cancer progression [68]. These findings highlight the potential application of non-coding RNAs in regulating STAT3 pathway.

#### Posttranslational modification in STAT3 activation

Although phosphorylation plays a crucial role in STAT3 activation, other posttranslational modifications, including acetylation, methylation and palmitoylation, also activate STAT3. As shown in Fig. 1a, several residues located in NTD, SH2 and TAD domains could be acetylated, mainly mediated by p300/CREB-binding protein (p300/CBP) acetyltransferase. For instance, STAT3 is acetylated by p300/CBP at Lys49 and Lys87 and therefore enhancing STAT3 transcriptional activity [69, 70]. Lys707 and Lys709 located in TAD domain are also acetylated by p300/CBP, which is required for STAT3 mitochondrial translocation and subsequent regulation of pyruvate metabolism [71]. Maupali et al. also demonstrated that a histone-modifying enzyme enhancer of zeste homolog 2 binds to and methylates STAT3 at Lys49 and Lys180, leading to enhanced STAT3 activity [72]. Residue Cys108 could be palmitoylated by DHHC7 (DHHC family member of palmitoyltransferases) and promotes STAT3 membrane recruitment and phosphorylation [73].

### Negative regulators of STAT3

In normal physiological conditions, the STAT3 activation is strictly regulated by some negative regulators to avoid excessive stimulation. The negative modulators are listed in Table S1.

#### SOCS proteins, PIAS proteins and protein tyrosine phosphatases block STAT3 activity

SOCS family members consist of SOCS1–SOCS7 and CIS, most but not all of which block the JAKs–STAT3 signaling. The regulatory mechanisms employed by members of the SOCS family in signaling modulation include: inhibition of STAT3 binding to activating receptors, suppression of JAKs, ubiquitination, and subsequent degradation of target proteins [74, 75]. SOCS1 and SOCS3, the most potent SOCS family members, display vital roles in inflammation and cancers. Reduced expression or mutation of SOCS1 and SOCS3 causes constitutive STAT3 activation, which accelerates the progression of pancreatic ductal adenocarcinoma [76], prostate cancer [77], glioblastoma [78]. Likewise, lack of SOCS2 regulates the inflammation and tumorigenesis mediated by STAT3 in hepatocellular carcinoma [79]. Higher expression of SOCS4 and SOCS7 associates with earlier tumor stage, more favorable OS and RFS in breast cancer [80]. Furthermore, lower expression levels of SOCS5 and SOCS6 correlate with poor prognosis in liver cancer [81], prostate cancer [82] and colorectal cancer [83]. PIAS family members consist of PIAS1–PIAS4, which modulate signaling by several mechanisms including the blockade of the DNA-binding ability of STAT3, recruitment of transcriptional co-repressors, and promoting protein SUMOylation [84]. PTPs (such as PTPRK, SHP1, SHP2, PTPN1, PTPN2, PTPN9) are a large family of phosphatases responsible for dephosphorylating tyrosine residues in phosphorylated proteins, and these enzymes play crucial roles in disrupting STAT3 activity [85].

#### Long non-coding RNAs and microRNAs block STAT3 activity

Alongside the oncogenic roles of lncRNAs and miRNAs, extensive studies have also revealed their tumor suppressor roles in diverse cancer types. According to recent findings, LINC00908-encoded polypeptide ASRPS directly binds to STAT3 at the coiled-coiled domain and reduces the activity of STAT3 [86]. Long intergenic non-coding RNA p21 functions as a tumor suppressor factor through directly blocking STAT3 activity and thus inhibits G1/S transition and induces apoptosis in head and neck squamous cell carcinoma (HNSCC) cells [87]. The main mechanisms of miRNA function as STAT3 suppressor are as follows: directly by sequence-complementary with STAT3 at 3′-UTR, 5′-UTR or the coding regions; indirectly blocking STAT3 activation through degrading the mRNAs of upstream regulators such as IL-6, IL-6R and JAKs. For example, miR-124, miR-125, miR-320, miR-1301 directly target 3′-UTR of STAT3 and inhibit cell cycle progression, proliferation, migration and invasion in bladder cancer [88], gastric cancer [89], lung adenocarcinoma and colorectal cancer [90, 91]. In addition, miR-9 and miR-26a directly target the 3′-UTR of IL-6 mRNA and therefore block IL-6-JAK2-STAT3 signaling [92, 93].

#### Posttranslational modification block STAT3 activity

Since the translational modification is reversible, the dynamic equilibrium of acetylation and deacetylation, methylation and demethylation are important for maintaining the activity of STAT3. As shown in Fig. 1a, the acetylated STAT3 at Lys615, Lys631 and Lys685 are deacetylated and deacetyliminated by lysyl oxidase 3, which suppresses the STAT3 dimerization, abolishes STAT3 transcriptional activity, and inhibits cell proliferation [94]. Methylation of STAT3 on K140 by the histone methyltransferase SET9 blocks the STAT3 activity in response to IL-6 [95]. Other translational modifications are also identified to block STAT3 activity. For example, AR degradation enhancer ASC-J9® suppresses prostate cancer cell invasion via modulating the STAT3 SUMOylation at Lys679 to alter the phosphorylation status of STAT3 [96]. S-Glutathionylation at Cys328 and Cys542 impairs STAT3 phosphorylation [97] and alkylation at Cys468 and thus inhibiting STAT3 DNA-binding ability [98]. Moreover, S-Nitrosylation of STAT3 at Cys259 disrupts the IL-6 induced STAT3 phosphorylation for genes required for inflammatory/immune responses and cell proliferation, including cancer [99].

## STAT3 in cancers

In this part, we mainly discuss how STAT3 mediates cancer hallmarks activities. Parts of target genes regulated by STAT3 are listed in Table S2.

### Proliferation and resistance to apoptosis

In keeping with the concept that cancer is a disease which occurs in the unbalanced status of persisting proliferation and resistance to apoptosis. There are considerable studies on STAT3 promoting cell proliferation, survival and resisting apoptosis. The main mechanisms of STAT3 regulating proliferation are due to the uncontrolled flux through the cell cycle. Cyclin D1, Pim1 and c-Myc are found involved in STAT3 mediating the abnormal G1/S phase transition [100, 101]. Moreover, STAT3 also participates in modulating G2–M phase checkpoint by up-regulating the expression of cyclin B1 and Cdc2 via early 2 factor [102].

The most extensively-elucidated mechanisms of STAT3 in mediating the apoptosis escape are: the constitutive activation of STAT3 promotes the expression of anti-apoptotic proteins Bcl-2 and Bcl-2-related family members such as BCL-XL, MCL-1 and inhibitor of apoptosis proteins (Inhibitor of apoptosis protein-2, survivin), while downregulating the Fas mediates intrinsic apoptotic pathway. Much evidence has shown that Bcl-2, BCL-XL, MCL-1 are highly expressed in tumors and correlate with histopathologic features, clinical progress and expression levels of phosphorylated STAT3 [103].

### Migration, invasion and angiogenesis

Cumulative pieces of evidence show that STAT3 plays critical roles in all steps of cancer metastasis including invasion, migration, and angiogenesis. Invasion to the extracellular matrix by regulating the matrix metalloproteinases (MMPs) is a prerequisite for metastasis of cancer cell [104]. IL-6/STAT3 upregulates the expression levels of MMPs including MMP-1, MMP-2, MMP-7, MMP-9 via directly interacting with their promotors in several aggressive cancers [105]. Moreover, the EMT also plays a central role during the initial metastasis. Considerable evidence has demonstrated that STAT3 induces the expression of EMT associated genes including TWIST, ZEB1/2, snail, vimentin, N-cadherin, and suppresses the expression of E-cadherin [106–108].

**Fig. 2:**
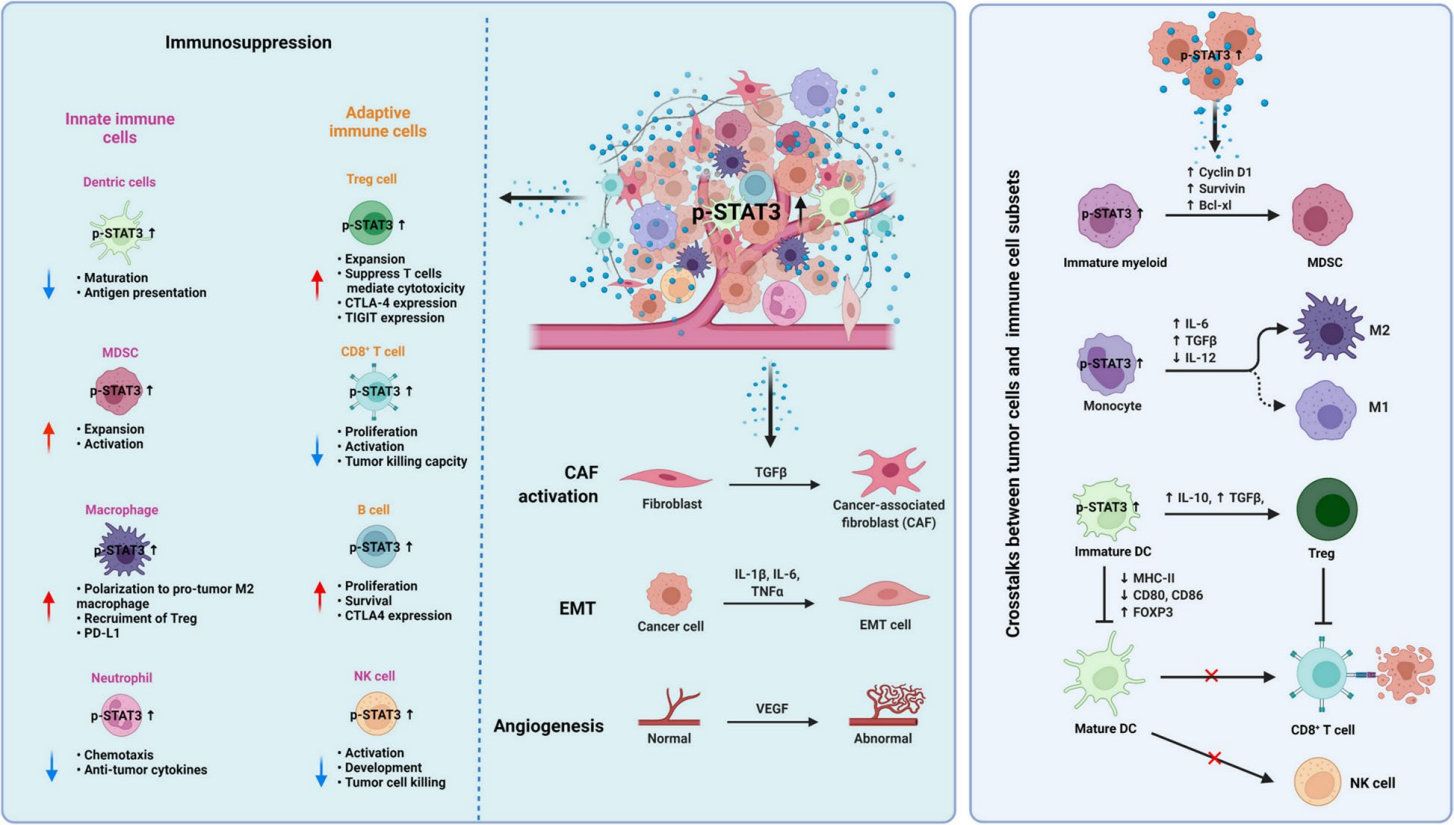
STAT3 regulates immunosuppression and the crosstalk between tumor cells and immune cells in TME. Left panel, STAT3 activation induces the immunosuppression of innate immune, adaptive immune cells, as well as tumor-promoting activities of fibroblasts and endothelial cells. STAT3 activation promotes the expansion and proliferation of immunosuppressive MDSC and B cells, and drives the expansion and pro-tumor M2 polarization of immunosuppressive Treg cells and macrophage cells. Moreover, STAT3 activation simultaneously induces the expression of immune checkpoint molecules including PD-1, TIGIT and CTLA-4 in these cells. In addition, STAT3 activation impairs the immune-associated antitumor activities of neutrophils, CD8^+^ T cells and NK cells. STAT3 activation also suppresses the antitumor activity of dendritic cells via disrupting the maturation and antigen presentation. Purple fonts represent the innate immune cell subsets, orange fonts represent adaptive immune cell subsets. Red arrows represent tumor promoting function and blue arrows represent the decreasing antitumor function. Right panel, STAT3 regulates the crosstalk between tumor cells and immune cells in TME. Increasing STAT3 activities in tumor cells promotes the production of IL-6, IL-10, VEGF, TGFβ. STAT3 activation and these cytokines and factors mediated the expansion and polarization of MDSC and M2 macrophage. The increasing STAT3 activities diminish the maturation of dendritic cells which leads to the accumulation of Treg cells and thus blocks the antitumor activities of CD8^+^ T and NK cells. Created with BioRender.com

Many studies have shown that STAT3 activation in tumor cells governs the secretion of diverse pro-inflammatory factors including IL-6, IL-10 and VEGF. Additionally, it diminishes the activity of natural killer cells, thereby facilitating immune evasion by tumor cells during circulation [109], as depicted in Fig. 2. After surviving from the circulation, the fast-growth of survival tumor cells metastasized in the distant organs requires a high demand for oxygen and nutrients. Thus, angiogenesis is essential for the tumor growth and metastasis. VEGF upregulation is mediated by excess STAT3 signaling in diverse human cancer cell lines [15]. Besides these, various studies reveal that STAT3 binds to the promotor of basic fibroblast growth factor (bFGF), a pro-angiogenic growth factor that activates FGFR and promotes endothelial cells angiogenesis [110].

### Tumor immunosuppression in tumor microenvironment (TME)

TME is a complex and heterogeneous system. Many tumor immunologists have shown that the cross-talk between STAT3 activation in tumor cells and other cell populations in TME, tightly controls the immune escape and pro-inflammation, thereby facilitating tumor progression [111]. Here we focus on the underlying mechanisms of STAT3 activation in tumor-infiltrating immune cells, which contribute to both innate and adaptive immune suppression, thereby impeding the antitumor efficacy of effector cells.

As shown in Fig. 2, the main mechanisms of immune escape and tolerance in TME are caused by dysfunction of dendritic cells (DCs), myeloid-derived suppressor cells (MDSCs), tumor-associated macrophages cells (TAMs) and tumor-associated neutrophils cells (TANs). Exhaustion of lymphocytes (T cells) and natural killer (NK) cells is mediated by inhibitory signals like cytokines (IL-6, IL-10) and growth factors (VEGF, TGFβ). The activation of immune checkpoint molecules like programmed death-1 (PD-1), T cell immunoglobulin and mucin domain-containing-3 (TIM-3), cytotoxic T-lymphocyte associated protein-4 (CTLA-4) [112], lymphocyte-activation gene 3 (LAG-3) [113], B7 homolog 3 protein (B7-H3) [114], T-cell immunoreceptor with Ig and ITIM domains (TIGIT) [115], serve as negative regulators of anti-tumor immune response.

Dendritic cells are the antigen presenting cells which activate tumor-specific T-cell responses. Yu’s group has previously demonstrated that STAT3 activation shuts down the innate immune-stimulating molecules such as interferon γ induced by CD8^+^ T cells, pro-inflammation cytokines (IL-12, tumor necrosis factor-α) and chemokines (C-C Motif Chemokine Ligand 5 (CCL5), C-C Motif Chemokine Ligand 9 (CCL9)), and suppresses the maturation of dendritic cells via secreting tumor-associated factors such as IL-6, IL-10, VEGF which in turn activate STAT3 in a positive feedback loop [111]. Increasing expression of STAT3 leads to the accumulation of immature dendritic cells [116]15. Accordingly, blocking the STAT3 activity in tumor cells leads to the maturation of dendritic cells [109].

The expansion and activation of MDSCs have a remarkable function in immune response suppression and enhancing tumor progression. STAT3 activation plays crucial roles in differentiation and expansion of MDSC [117]. Gabrilovich DI’s group reported that STAT3 potentially regulates the survival and proliferation of myeloid progenitor cells and prevents them from differentiating into mature myeloid cells via upregulating cyclin D1, survivin, BCL-XL, thereby inducing immune suppression [118].

Tumor-associated macrophage cells represent the predominant immune cell population in TME and exert pivotal roles in promoting tumor growth, angiogenesis, drug resistance and immunosuppression [119, 120]. Recently, studies have found that IL-6 promotes normal macrophages to differentiate into M2 macrophages mediated by STAT3 and therefore supports tumor proliferation in gastric cancer [121]. Moreover, M2-polarized macrophages convert to the anti-tumor M1 type when STAT3 signaling is disrupted [122]. The IL-27/STAT3 signaling axis also induces the expression of immune checkpoint molecules including programmed death-1 (PD-1) and programmed death-2 (PD-2) in macrophages in lymphoma [112].

Tumor-associated neutrophil cells are also one of the most infiltrating immune cells within the tumors. High expression of STAT3 in neutrophils attenuates their tumor-killing activities [123]. Since IFN-β suppresses the activity of STAT3, IFN-β–deficient mice display more neutrophils with higher levels of VEGF and MMP9, both regulated by STAT3, thus promoting tumor growth [124]. The cancer-associated fibroblasts (CAF) protect the PD-L1^+^ neutrophils from apoptosis and foster immune suppression through the IL6-STAT3 pathway in hepatocellular carcinoma [125]. Moreover, neutrophils enhance the migration, invasion and EMT of gastric cancer cells through the IL-17a/JAK2/STAT3 signaling, meanwhile blocking the IL-7a or disrupting the JAK2/STAT3 signaling increase the tumor cytotoxicity of neutrophils against the cancer [126].

T regulatory cells (Treg cells) are a unique subpopulation of CD4^+^ T cells which inhibit T cell-mediated cytotoxicity or produce the soluble immunosuppressive molecules including IL-10, TGFβ, and adenosine [127]. In cancer patients, high numbers of Treg cells are associated with lymph nodes, histological grade, and TNM stage [128]. One study demonstrates that STAT3 is a transcription cofactor for FOXP3 and maintains the phenotype and function of Treg cells. Ablating STAT3 suppresses the expression of FOXP3 and therefore inhibits the function of CD25^+^CD4^+^ Treg cells [127]. Evidence has shown that activation of STAT3 stimulated by IL-10 and TGFβ in tumor-infiltrating DCs impedes CD8^+^ T cell function, and contributes to accumulation and proliferation of tolerogenic Treg cells inside tumors [123]. Moreover, CTLA-4 is constitutively expressed on CD25^+^CD4^+^ Treg cells and thus contributes to maintaining its immunologic self-tolerance [129].

CD8^+^ T cells (cytotoxic T cells, Tc) play a pivotal role in exerting positive immune control over cancer progression. STAT3 is reported to inhibit the CD8^+^ T cells accumulation in tumor and thus inhibiting the immune response through downregulating CXCR3/CXCL10 axis [130]. Ablating STAT3 in engineering CD8^+^ T cells results in enhanced tumor antigen-specific T cell activity and tumor growth inhibition [131]. Another group has demonstrated that one STAT3-blocked whole-cell vaccine impairs the TIGIT expression in the CD8^+^ T cells [132]. As previously mentioned, CD8^+^ T cells show crucial roles in Treg cells mediated immune suppression. *P*-STAT3 is upregulated in circulating CD8^+^ T cells and is associated with elevated levels of IL-4, IL-6 and IL-10 as well as reduced level of interferon γ, therefore contributing to the pathogenesis of HCC [133].

Extensive studies have demonstrated that B cells have yin and yang roles in tumor development and progression [134]. Meanwhile, STAT3 activation in B cells promotes the angiogenesis in melanoma and lung cancer [135]. Moreover, increased B cell infiltration and *p*-STAT3 expression in cancers are associated with poorer survival [136]. Furthermore, Herrmann and co-workers have demonstrated that the CTLA4/Tyk2/STAT3 axis is critical to the proliferation and survival of B cells and thus leads to the immune suppression in melanoma and lymphoma [137].

Natural killer cells mediate the innate defense to protect the host against viral infection and the progression of cancerous cells. NK cells also have critical roles in regulating the activity of T cells, DCs, neutrophils, macrophages in TME. Nicholas has given a systemic summary of STAT3’s negative functions on NK cell biology, including NK development, activation, target cell killing, and fine-tuning of the innate and adaptive immune responses [138].

Collectively, STAT3 plays leading roles in the immunoediting from immune surveillance to immune escapes in the microenvironment. Therefore, a novel therapeutic approach involving the targeted inhibition of STAT3 combined with the restoration of aberrant immune escape mechanisms within the tumor microenvironment represents a promising strategy that has gained significant traction in ongoing clinical trials.

### Reprogramming metabolisms

**Fig. 3:**
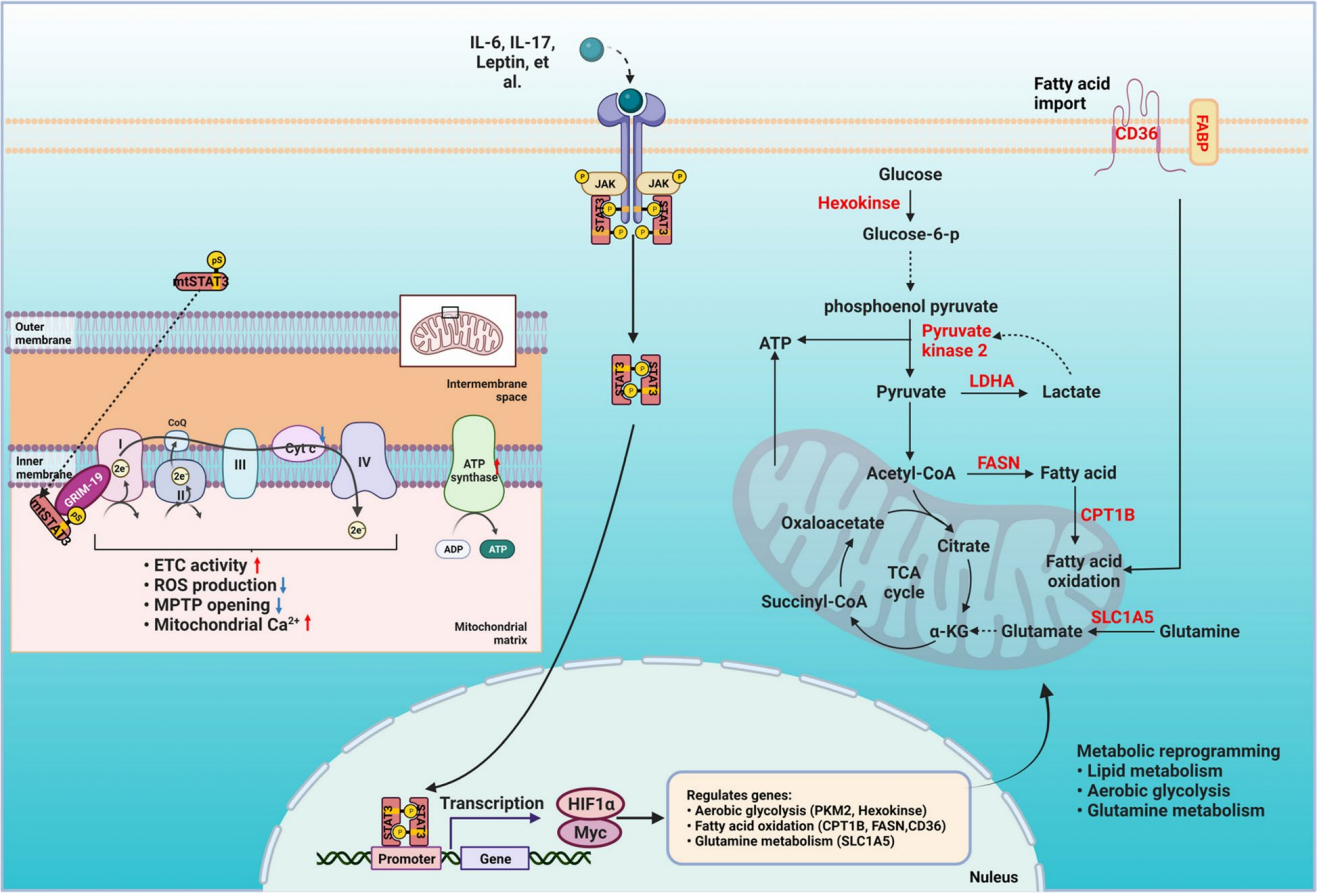
STAT3 regulates the aerobic glycolysis, lipid metabolism, glutamine metabolism and energy production in tumor cells. The STAT3 activation regulates these metabolic reprogramming through the transcriptional regulation of genes involved in these processes, which contribute to multiple hallmarks of cancer including proliferation, immunosuppression, cancer stemness and drug resistance. Moreover, the mtSTAT3 accelerates ETC activity, decreases ROS production and MPTP opening, and promotes the calcium retention via binding to GRIM-19, which provides privileged advantages for the cancer progression. Created with BioRender.com

Apart from the canonical activities of STAT3 in pleiotropic effects on a spectrum of tumor processes, STAT3 also functions as a hub for energy and matter metabolism via its different subcellular activities such as nuclear, mitochondrial, and cytoplasmic STAT3 activities. As shown in Fig. 3, we emphasize the functions of mitochondrial STAT3 on enhancing the activity of the ETC, stimulating the ATP synthesis, restricting ROS production, maintaining mitochondrial permeability transition pore (MPTP), and promoting mitochondrial Ca^2+^ influx [139]. We also highlight that STAT3 signaling regulates the Warburg effects [71], fatty acid oxidation [140], and amino acid metabolism [141] of cancer cells. In these ways, STAT3 shapes the metabolism to provide more favorable energy and metabolic intermediates for rapid tumor growth in the conditions of metabolic stress.

Mitochondrial STAT3 (mtSTAT3) is required for optimal function of mitochondria [25, 142]. mtSTAT3 directly incorporates into complex I located in the inner mitochondrial membrane via specifically binding to the retinoid-interferon-induced mortality (GRIM-19), an integral component of complex I, enhancing the electron transfer through complex I, II, III, IV and V of ETC, promoting the ATP synthase activity and thus attenuating the ROS production [143]. The mtSTAT3 opens a new area of STAT3 tumor-promoting roles in cancer research. The accelerating mitochondrial respiration, decreasing ROS production and calcium retention triggered by mtSTAT3 provide privileged advantages for the cancer progression [26].

Recently, mtSTAT3 is also reported to regulate the immune response. mtSTAT3 enhances mitochondrial Ca^2+^ mediated motility of CD4^+^ T cells which is essential for them to find and reach their targets and mediate pathogenesis [144]. mtSTAT3 may participate in CD8^+^ T cell memory enhanced by IL-21 and the production of antibody mediates by IL-21 in B cells [145]. Moreover, the conventional STAT3 transcriptional function in CD4^+^ T cells differentiating into Th17 cells is later complimented by mtSTAT3 sustains pathogenic Th17 cell proliferation and cytokine response to antigen [146]. One group also identified that mitochondrial oxidative phosphorylation regulates the fate decision between pathogenic Th17 and regulatory T cells which requires the participation of mtSTAT3 [147].

STAT3 mediates the Warburg effect by enhancing aerobic glycolysis and reducing oxidative phosphorylation even in the presence of oxygen in several cancer types. The high expression of HIF-1α and Myc mediated by STAT3 commonly play key roles in this metabolic switch [148]. Furthermore, STAT3 regulates aerobic glycolysis by stimulating hexokinase 2 in breast cancer [149], and upregulating lactate dehydrogenase A (LDHA) in myeloma [150]. PKM2, a key player of the Warburg effect which is induced by HIF-1α, and STAT3 acts as HIF-1α/PKM2 feed-back loop participants which in turn enhances the Warburg effect in cancer [151, 152].

Recently, the crosstalk between lipid metabolism and tumorigenesis mediated by STAT3 are observed through the cytokines, hormones and adipokines, which paracrine or endocrine secreted by the adipose tissues around the tumor cells. For example, leptin, an adipokine, stimulates STAT3 activation, and promotes the fatty acid oxidation (FAO) via inducing the expression of carnitine palmitoyltransferase 1B (CPT1B), which finally resulted in the inhibition of CD8^+^ T effector cell glycolysis and promotion the progression of breast tumor [20]. Furthermore, the JAK/STAT3 mediates enhancement of FAO, thereby promoting the cancer stem cell self-renewal and chemoresistance in several cancer types including breast cancer [153], lung cancer [154] and gastric cancer [155]. STAT3 also upregulates the expression of fatty acid synthase (FASN), which mediates de novo fatty acid synthesis [156]. Several studies have demonstrated that FASN is commonly upregulated in malignant tumor and promotes the tumor progression [157]. In addition, the pro-inflammatory cytokine IL-17A promotes the expression of STAT3 mediating fatty acids binding protein 4 (FABP4), which acts synergistically with fatty acid receptor CD36 to initiate the uptake of fatty acids to fuel ovarian cancer growth [158]. One group recently reported that the lipid metabolic regulators such as SREBP1, fatty acid transporter CD36, and FABP6 influence the metastatic potential of cancer cells [159]. All of these genes display co-dependency with STAT3 transcription [160, 161]. Targeting of CD36 with neutralizing antibodies impairs metastasis in human melanoma, breast and oral cancers [162]. Thus, the STAT3 mediates lipid metabolism may drive the metastatic potential of malignancies.

STAT3 also regulates amino acid metabolism. Many cancer cells are addicted to glutamine, which serves to generate peptides (including glutathione) and proteins which contribute to cancer progression. Recently, one group demonstrated that STAT3 regulates glutaminolysis and amino acid (glutamine, glutathione) influx as the anaplerotic reactions to the TCA cycle in leukemia stem cells by promoting expression of MYC, which in turn regulates the transcription of SLC1A5 [141]. Consequently, dual-inhibition of glutamine entry into the tricarboxylic acid cycle (TCA cycle) and STAT3 signaling provide a promising therapeutic strategy for ovarian cancer [163].

### Cancer stemness maintenance

Cancer stemness cells (CSCs) are defined as the self-renewing cancer cells that promote tumor initiation, metastasis, relapse, and therapeutic resistance. The main ways by which STAT3 modulates stemness are through mediation of EMT and the immunity program [164]. STAT3 activation induces the EMT and promotes generation of CSCs through upregulating EMT associated transcription factors including Snail, Zeb1, JUNB, and Twist-1 or Nanog/slug axis [165]. Accordingly, blocking the EMT is a promising strategy for CSC targeting. Furthermore, STAT3 is reported to regulate the CSCs markers like CD24, CD34, CD44, CD90, CD133 and aldehyde dehydrogenase (ALDH) [166, 167]. More recent evidence indicates that immune cells from the microenvironment contribute to the phenotype of CSCs [168]. For example, TAMs promote the CSC-like phenotypes in breast cancer via activating a paracrine EGFR/STAT3/SOX-2 signaling pathway [169]. Paracrine-derived IL-8 and GRO chemokines secreted by the M2 macrophages in inflammatory breast cancer promote mesenchymal and CSC-like phenotypes [170].

### Chemoresistance

The main mechanisms of STAT3 in regulation of chemoresistance are due to the extensive cancer hallmark features of STAT3, the feedback loops or altered crosstalk between STAT3 and other signaling pathways. For instance, the STAT3/OcT-4/c-Myc axis facilitates the enrichment of CSCs, which drives the triple-negative breast cancer cells’ resistance to doxorubicin treatment [21]. Furthermore, the feedback activation of STAT3 promotes secondary drug resistance in TKI therapy (blocking EGFR, FGFR, MEKs, HER2, ALK, MET, KRAS), chemotherapy or radiotherapy. This acquired resistance is generally induced by the mutation, epigenetic modification or abnormal expression of the drug target gene, which promote parallel activation of STAT3 pathways [171]. The reprogramming of TME such as hypoxia, inflammatory cytokines, abnormal pH, or altered crosstalk between tumor cells and microenvironment as disease progresses also accelerates a secondary STAT3 activation following primary therapy [172, 173]. For example, the cancer-associated fibroblasts treated with cisplatin simulated IL-11 upregulated and subsequently activated STAT3, which finally promotes the lung adenocarcinoma cell resistance to cisplatin [174]. MEK inhibition leads to autocrine activation of STAT3 via the parallel activation of the second RTKs like FGFR and JAKs [175]. Y-box binding protein-1 was initially found to enhance drug resistance via upregulating ATP-binding cassette (ABC) transporters members and eventually leading to the upregulation of *P*-glycoprotein [176]. Taken together, these connections provide possible compensatory mechanisms, allowing cells to respond more adaptively to the dynamic environment.

### STAT3 and exosome mediates tumor hallmarks’ activities

Exosome is a subset of extracellular vesicles secreted by most eukaryotic cells. As the rapid advances in exosome mediates biological activities, many studies suggest that exosome tightly crosstalk with STAT3-mediated cancer hallmark features. The exosome p120-catenin inhibits the liver cancer cells proliferation via STAT3. Meanwhile, blocking STAT3 by inhibitor abolishes the tumor-suppressive function of exosome p120-catenin [22]. Hypoxic bone marrow-derived mesenchymal stem cells derived exosome mediates transfer of several miRNAs that promote metastasis of lung cancer cells via STAT3-induced EMT [177]. Furthermore, *p*-STAT3-containing exosomes contribute to acquired 5-FU resistance in colorectal cancer [178]. Glioblastoma stem cells derived exosomes release STAT3 and facilitate the accumulation of PD-L1 and M2 macrophage which eventually trigger the immunosuppressive microenvironment [179]. Exosome-based inhibitors open up new horizons for STAT3 drug discovery. Chuang and co-workers found a novel STAT3 inhibitor, pacritinib, which overcomes temozolomide resistance via downregulating miR-21-enriched exosomes from M2 glioblastoma-associated macrophages [180]. Accordingly, targeting STAT3 provides a basis for using exosomes to serve as a strategy in clinical therapeutics.

## Pharmacological agents targeting STAT3

**Fig. 4:**
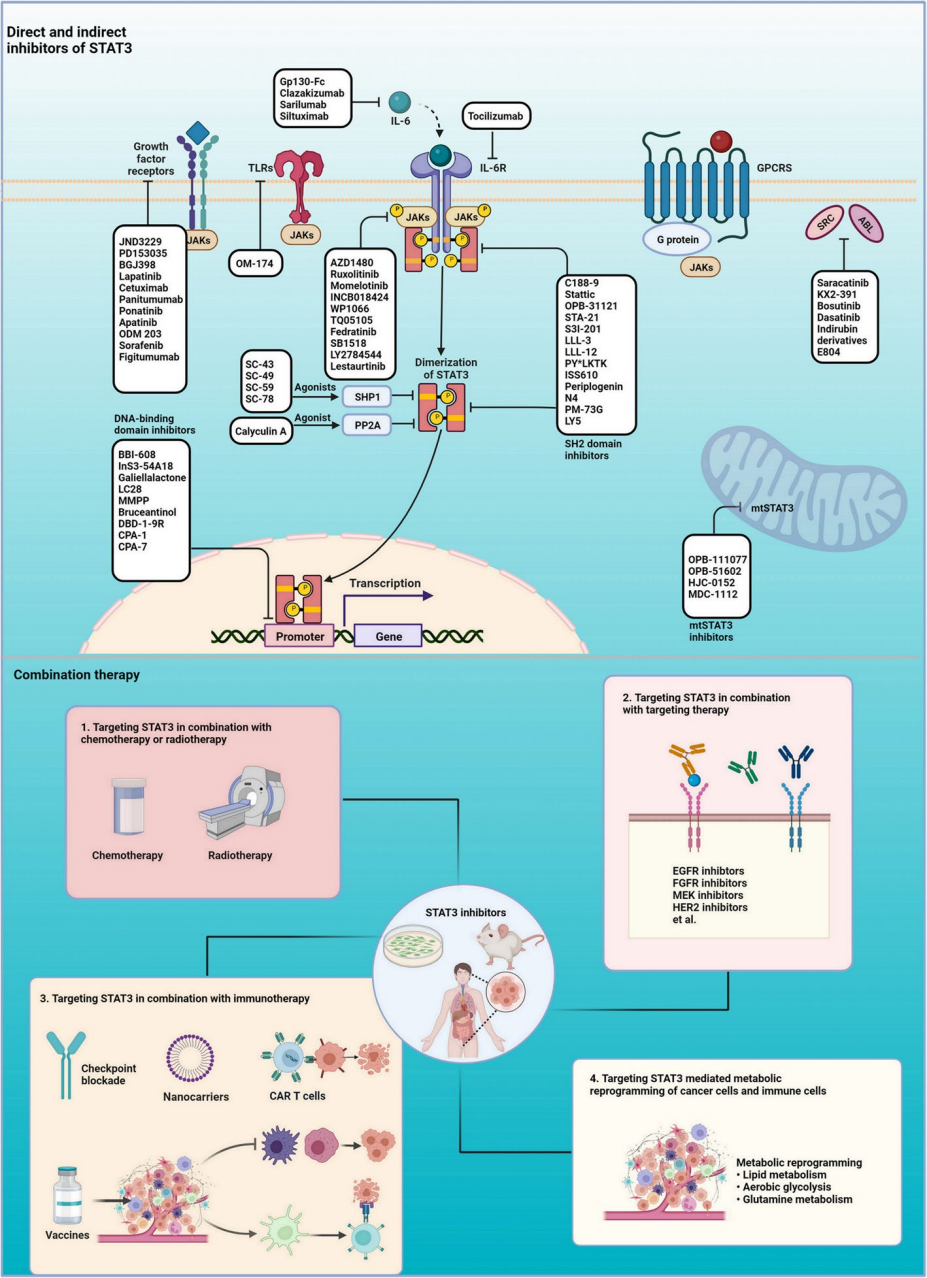
Summary of STAT3 direct inhibitors and indirect inhibitors and the combination strategies. Upper panel, an overview of STAT3 direct inhibitors and indirect inhibitors. Lower panel, STAT3 inhibitors in combination with chemotherapy, radiotherapy, targeted therapy, immunotherapy, and dual-inhibiting of STAT3 mediate metabolic alteration of tumor cells and immune cells. Created with BioRender.com

In the past three decades, many attempts have made aims at developing the selective and potent STAT3 inhibitors. In the following section, we will emphasize the direct and indirect STAT3 inhibitors relevant to pre-clinical development and clinical trials (as shown in Fig. 4). The STAT3 inhibitors are including but not limited to those listed in Tables 2 and 3. The cell-based efficacy, the administration of animal models and the information of clinical trials are provided. Due to the comprehensive reviews that have previously covered this topic, we mainly focus on discussing the latest research and the novel insights on STAT3 inhibitors.

**Table 2:** Inhibitors of STAT3 in pre-clinical studies

Type	Drug names	Cell-based efficacy (IC50/EC50/GI50)	Animal models (Types, administration)	Ref
**Direct inhibitors**				
**NTD**	ST3-H2A2 (Peptide)	The IC50 of prostate cancer cells (LNCaP, PC3, DU145) were less than 5 μM.	NA	[181]
**CCD**	MS3–6 (Ab)	NA	NA	[182]
**DBD**	InS3-54A18 (SM)	Lung cancer cells (A549, H1299) and breast cancer cells (MDA-MB-231, MDA-MB-468), 3.2–4.7 μM.	A549 xenograft, 200 mg/kg, oral dosing 2–3 times a week for 4 weeks.	[183]
	Galiellalactone (SM)	Docetaxel-resistant DU145 cells spheres (6.2 μM), docetaxel-sensitive DU145 spheres (10.1 μM)	DU145 xenograft, 1 mg/kg and 3 mg/kg, i.p injection QD for 3 weeks.	[184, 185]
	SG-1709 (SM)	MDA–MB-468 (10.14 μM)	NA	[186]
	SG-1721 (SM)	MDA–MB-468 (6.9 μM)	0.5 mg/kg, i.p 3 times/week for 20 days	[186]
	GPA512 (SM)	DU145 (4.81 mM)	Orally, DU145 bearing nude mice, 40 mg/kg, QD five times/week.	[187]
	HJC0152 (SM)	AGS and MKN45, less than 10 μM.	MKN45 bearing BALB/c nude mice, 7.5 mg/kg HJC0152 dissolved in 100 μL PBS i.p twice weekly for 21 days.	[188]
	Silibinin (SM)	MDA-MB-231, 200―250 μM.	Female SKH-1 hairless mice, 1% (w/w) silibinin fed in diet, less tumor numbers in UVB caused tumorigenesis.	[189, 190]
	HO-3876 (SM)	NA	A2780 bearing BALB/c nude mice, HO-3867 was mixed with the animal feed (Harlan Teklad) at 3 different levels (25, 50, and 100 ppm).	[191]
	LC28 (SM)	H1299 (8.1 ± 4.1 μM), SKOV3 (3.7 ± 0.7 μM), SKOV3/DDP (14.7 ± 2.4 μM)	NA	[192]
	MMPP (SM)	NCI-H460 (1.95 μg/mL), NSCLC (12.3 μg/mL)	NCI-H460 xenograft model and PDX model, 2.5–5 mg/kg, i.p. twice a week for 3 weeks.	[193]
	Bruceantinol (SM)	HCT116 (18.7 ± 3.3 nM), HCT116 p53^−/−^(18.7 ± 3.3 nM), HCA-7 (23.6 ± 4.8 nM), H630 (43.2 ± 5.9 nM), H630R1 (49.7 ± 4.6 nM)	HCT116 tumor bearing xenografts, 2 mg/kg and 4 mg/kg; i.p. thrice per week.	[194]
	DBD-1-9R (Peptide)	U266 cells, 270 nM	NA	[195]
	15-mer duplex ODN (Oligonucleotides)	SCCHN Cell, less than 25 μM	NA	[196]
	CPA-7 (Platinum-based)	RM-9 cells, 20 μM	0.75 mg/kg, 1.5 mg/kg, RM-9 tumor bearing C57BL/6 J mice, tail vein injection every three days	[197]
**Linker domain**	BPMB (SM)	NA	NA	[198]
**SH2 domain**	Periplogenin (SM)	KYSE30 (1.707 ± 0.275 μM); KYSE70 (2.898 ± 0.959 μM); KYSE450 (1.201 ± 0.167 μM).	ESCC patient-derived xenograft, i.p BID, 8 mg/kg and 16 mg/kg.	[33]
	ODZ10117 (SM)	NA	GSC528 tumor bearing BALB/c nu/nu nude mice, i.p injection, 0.1 mg/kg or 1 mg/kg, six times per week; U87-MG tumor bearing BALB/c nu/nu nude mice, BID for 2 weeks.	[199]
	N4 (SM)	CAPAN-2, PANC-1, MIAPACA-2, BXPC-3, HPAC, CFPAC-1, less than 4 μM.	PANC-1 cells tumor bearing BALB/c-nude mice, i.p QD for 20 days.	[200]
	SD-36 (SM)	MOLM-16 (35 nM), DEL (1.48 μM), Karpas (0.98 μM), KI-JK (0.18 μM), SU-DHL-1 (0.25 μM)	SCID mice bearing MOLM-16 tumors; 100 mg/kg weekly; 50 mg/kg 3 times per week; 100 mg/kg twice a week for 3 weeks.	[201]
	Stattic (SM)	OSC-19 (3.481 ± 0.953 μM), UM-SCC-17B (2.562 ± 0.409 μM), Cal33 (2.282 ± 0.423 μM), UM-SCC-22B (2.648 ± 0.542 μM)	UM-SCC-17B tumor-bearing mice, 50 mg/kg, orally gavage, 5 days a week for 4 weeks.	[202]
	S3I-201 (SM)	LNCaP (300 μM)	Human breast (MDA-MB-231) tumor-bearing mice were given S3I-201 (5 mg/kg) i.v. every 2 or every 3 days.	[203, 204]
	S3I-1757 (SM)	MDA-MB-468, MDA-MB-231, H358, A549 (less than 200 μM).	B16-F10 melanoma xenograft, daily i.v injections of PBS (control) (*n* = 3), free S3I-1757 (*n* = 3), or S3I-1757 encapsulated in PEO114-b-PBCL20 micelles (*n* = 3) with a dose of 1 mg/kg for 7 days.	[205, 206]
	STA-21 (SM)	DU145 (12.2 μM)	NA	[207]
	LLL-3 (SM)	Glioblastoma cell U87, U251, U373 (less than 30 μM)	U87 intracranial tumor-brearing athymic nude mice (nu/nu), i.v injection of 50 mg kg − 1 of LLL-3 administered stereotaxically.	[208]
	LLL-12 (SM)	HCC cells, SNU387 (0.84 ± 0.23 μM), SNU398 (0.96 ± 0.18 μM), SNU449 (4.38 ± 1.25 μM), and Hep3B (2.39 ± 0.68 μM).	SNU398 tumor-bearing athymic nude mice, 5 mg/kg, i.p injection QD for one month.	[209]
	LY5 (SM)	1.637 to 3.347 μmol/L in liver cancer cells, 1.235 to 1.690 μmol/L in colon cancer cells	Colon cancer cell HCT116, QD i.p dosages of 5 mg/kg of LY5 or vehicle control for 11 days.	[210]
	SPI (Peptide)	MDA-MB-231, MDA-MB-435), Colo-357, DU145, A549, less than 80 μM.	NA	[211]
	PY*LKTK (Peptide)	NIH 3 T3/vSrc colony (more than 500 μM)	NA	[212]
	ISS610 (PM)	NIH 3 T3/vSrc colony (more than 1000 μM)	NA	[212]
	CJ-1383 (PM)	MDA-MB-231 (11.2 μM), MDA-MB-468 (3.6 μM).	NA	[213]
	PM-73G (PM)	MDA-MB-468, A549, more than 30 μM.	Orthotopic MDA-MB-468 breast tumor xenografts, intratumorally (i.t) with 100 μl of PM-73G, formulated in 20% hydroxypropyl-β-cyclodextrin (Trappsol) in PBS to facilitate solubility, 20 days.	[214, 215]
	T40214 (STAT3-G-Quartet)	PC3, 5 μM.	MDA-MD-468 or PC-3 tumor bearing athymic nude mice Balb/nu/nu, T40214 (5.0 mg/kg) plus polyethyleneimine (2.5 mg/kg), tail-vein injection, BID.	[216]
	T40231 (STAT3-G-Quartet)	PC3, 49 μM	MDA-MD-468 or PC-3 tumor bearing athymic nude mice Balb/nu/nu, T40231 (5.0 mg/kg) plus polyethyleneimine (2.5 mg/kg), tail-vein injection, BID.	[216]
**Other modes**	STAT3-IN-1 (SM)	IC50 of 1.82 μM and 2.14 μM in HT29 and MDA-MB 231 cells, respectively	Orally, 4 T1 mouse xenograft 10 mg/kg, 20 mg/kg.	[217]
	Cyclic STAT3 decoy (DecoyOligodeoxynucleotide)	IC50 of NSCLC is approximately 0.3 μM, the normal lung fibroblasts cell is 300 μM.	NSCLC xenograft, i.v injection, QD, 5 mg/kg/d.	[218]
	CTLA4apt-STAT3 (Aptamer)	NA	Karpas299 T cell lymphoma engrafted into athymic nude mice, 782.5 pmol/dose/mouse, i.v BID	[219]
**Indirect inhibitors**				
**IL-6/IL-6R**	Gp130-Fc (Ab)	NA	Colo357 (orthotopic pancreatic tumors cell) in SCID/bg mice, 0.5 mg/kg, 2.5 mg/kg, i.p. twice a week.	[220, 221]
**SRC**	Indirubin derivatives E804 (SM)	NA	NA	[222]
**EGFR**	JND3229 (SM)	BaF3, 0.51 ± 0.08 μM (L858R/T790M/C797S), NCI-H1975, 0.31 ± 0.01 μM (L858R/T790M), A431, 0.27 ± 0.18 μM (WT).	NA	[223]
	PD153035 (SM)	Human oral squamous cell carcinoma cells KB, KB/V, Hep2, A83, Tca83 and Tca8113, 2.57 ± 0.65, 0.73 ± 0.13, 7.71 ± 0.39, 9.35 ± 0.33, 7.14 ± 0.47 and 10.02 ± 0.28 μmol/l, respectively.	KB, KB/V bearing BALB/c nude mice, 4 or 8 mg/kg, QD i.p injection.	[224]
**FGFR**	BGJ398 (SM)	SKOV3ip1, less than 4 μM.	NA	[225]
**JAK1/2**	INCB16562 (SM)	TF-1 (102 ± 36 nM), Bcr-Abl-transduced TF-1 cells (more than 4000 nM).	NA-6.Tu1 xenografts, orally, 25 mg/kg, BID, 42 days.	[226]
	CIMO (SM)	HepG2, 7.3 μM.	Huh 7-Luc cells orthotopically athymic nu/nu female mice, 2 mg/kg, 10 mg/kg, 5 days a week i.p.	[227]
**JAK2**	TG101209 (SM)	Raji (8.18 μmol/L), Ramos (7.23 μmol/L), primary BL cells (4.57 μmol/L)	Ramos-derived tumor xenografts, orally gavage, 100 mg/kg, 7 consecutive days (*n* = 6 per group).	[228]
	TG101348 (SM)	HEL (305 nM), Ba/F3JAK2V617F (270 nM)	NA	[229]
	ZT55 (SM)	MCF-7, BT549, K562, KCL-22, U266, HEL, TF-1, cells (18.05—88.31 μM) Jurkat, Raji, NB4, and Molt-4 (more than 100 μM).	HEL xenograft tumors, athymic BALB/c nude mice, 100 mg/kg body weight, orally QD for 12 successive days.	[230]
	Compound 9# (SM)	B16F10 and 4 T1, little effect.	Tumor-bearing mice, i.t injection, 20 mg/kg, 40 mg/kg.	[231]
	BMS-911543 (SM)	SET2, A549, MDA-MB-231, MiaPaCa-2, 0.06—4.7 μM	BALB/c mice were immunized with KLH antigen followed by oral administration of BMS-911543 for 14 days at 3, 10 or 30 mg/kg QD.	[232]
**TYK2**	Cirsiliol (SM)	NA	ESCC patient-derived xenograft, SCID ice, gavage QD, 10 mg/kg and 50 mg/kg.	[233]
	NDI-031301 (SM)	DU.528, KOPT-K1, HPB-ALL, SKW-3, less than 10 μmol/l	KOPT-K1 cells engrafted into NSG mice, 100 mg/kg BID for 29 days.	[234]
**SHP1**	SC-59 (SM)	NA	6 Huh-7 cells bearing male NCr athymic nude mice, 10 mg/kg/day, orally, QD.	[235]
	SC-78 (SM)	HCT-116 (4.2 ± 0.4), HT-29 (4.1 ± 0.3)	NA	[236]
	SC-49 (SM)	NA	PLC5 (HCC) bearing NCr athymic nude mice, 5 mg/kg orally QD.	[237]
**PP2A**	Calyculin A (SM)	NA	NA	[238]

*SM* Small molecule, *Ab* Antibody, *PM* Peptidomimetic, *IC50* Half-maximal inhibitory concentration, *EC50* Half maximal effective concentration, *GI50* Growth inhibition by 50%, *i.p* Intraperitoneal injection, *i.v* Intravenous, *i.t* Intratumorally, *BID* Twice a day, *QD* Once a day, *NA* Not available

**Table 3:** Inhibitors of STAT3 in clinical development

Type	Drug names	Phase of trial	Status	Clinical Trial ID	Patients	Ref
**Direct inhibitors**						
**SH2 domain**	OPB-51602 (Small molecule)	I	Terminated	NCT02058017	Nasopharyngeal carcinoma (*n* = 9)	NA
		I	Completed	NCT01423903	Advanced cancer (*n* = 45)	NA
		I	Completed	NCT01344876	Hematological malignancies (*n* = 20)	[239]
		I	Completed	NCT01184807	Malignant solid tumor (*n* = 51)	[240]
	OPB-31121 (Small molecule)	I	Completed	NCT00955812	Advanced solid tumors (*n* = 30)	[241]
		I	Unkonwn	NCT00657176	Advanced solid tumors (*n* = 25)	[242]
	C188–9 (Small molecule)	I	Recruiting	NCT03195699	BC, HNSCC, NSCLC, CRC, melanoma, GAC, advanced cancer (*n* = 60)	NA
**DNA-binding domain**	BBI-608 (Napabucasin, small molecule)	I	Completed	NCT01775423	Advanced malignancies (*n* = 87)	NA
		I	Completed	NCT03525405	Healthy volunteers (*n* = 8)	[243]
		III	Completed	NCT01830621	CRC (*n* = 282)	[244]
		I/II	Completed	NCT02024607	GC (*n* = 495)	NA
		III	Completed	NCT02993731	Pancreatic cancer (*n* = 1132)	[245]
		I/II	Terminated	NCT02851004	Colorectal cancer (*n* = 55)	[246]
**Other modes**	AZD9150(Antisense oligonucleotide)	I	Completed	NCT01839604	HCC (*n* = 58)	NA
		I/II	Completed	NCT01563302	Lymphoma (*n* = 64)	[247]
		I	Completed	NA	Lymphoma, Lung Cancer (*n* = 25)	[248]
	STAT3 DECOY (Oligonucleotide)	I	Completed	NCT00696176	HNSCC (*n* = 32)	NA
	CpG-STAT3 siRNA	I	Recruiting	NCT04995536	Relapsed/Refractory B-Cell NHL (*n* = 18)	NA
	Pyrimethamine (Small molecule)	NA	Completed	NA	CLL (*n* = 16)	[249]
		I/II	Recruiting	NCT01066663	CLL, SLL (*n* = 26)	NA
	OPB-111077 (Small molecule)	I	Completed	NCT01711034	Advanced cancers (*n* = 145)	NA
		I	Terminated	NCT01942083	HCC (*n* = 33)	[250]
	Celecoxib (Small molecule)	III	Terminated	NCT00087256	CRC (*n* = 18)	NA
**Indirect inhibitors**						
**IL-6**	Siltuximab (Anti-IL-6 mAb)	I	Completed	NCT01219010	SMM, IMM (*n* = 30)	NA
		I	Completed	NCT00401765	Prostate cancer (*n* = 40)	NA
		I/II	Completed	NCT00265135	Metastatic RCC (*n* = 68)	[251]
		I/II	Completed	NCT00841191	Solid tumors (*n* = 106)	[252]
		II	Completed	NCT00402181	Multiple myeloma (*n* = 53)	[253]
		II	Completed	NCT00433446	Prostate cancer (*n* = 62)	[254]
		II	Terminated	NCT00385827	Prostate cancer (*n* = 106)	[255]
**IL-6R**	Tocilizumab (Anti-IL-6R mAb)	I	Terminated	NCT02336048	B-cell CLL (*n* = 38)	NA
		I	Completed	NCT03135171	Brest cancer (*n* = 11)	NA
		I/II	Completed	NCT01637532	Ovarian cancer (*n* = 21)	[256]
**SRC**	Saracatinib	II	Terminated	NCT00752206	Osteosarcoma (*n* = 38)	[257]
		II	Completed	NCT00669019	Melanoma (*n* = 23)	NA
	KX2–391	II	Completed	NCT01074138	Prostate cancer (*n* = 31)	NA
**SRC, ABL**	Bosutinib	III	Completed	NCT02130557	Leukemia, Myelogenous, Chronic, Breakpoint cluster region-abelson proto-oncogene (BCR-ABL) positive (*n* = 536)	[258]
	Dasatinib	II	Completed	NCT00439270	Prostate cancer (*n* = 49)	[259]
		III	Completed	NCT00744497	Prostate cancer (*n* = 1930)	[260]
		IV	Completed	NCT01660906	Chronic Phase Chronic Myeloid Leukemia (*n* = 39)	[261]
**EGFR**	Lapatinib	II	Completed	NCT00105950	Neoplasms, breast (*n* = 126)	[262]
	Cetuximab	II	Completed	NCT00084318	Head and neck cancer (*n* = 238)	NA
	Panitumumab	IV	Recruiting	NCT02301962	CRC (*n* = 58)	NA
**FGFR**	Ponatinib	II	Recruiting	NCT04043676	CML (*n* = 40)	NA
**VEGFR**	Apatinib	II	Unknown	NCT03709953	Lung cancer (*n* = 33)	NA
**FGFR/VEGFR**	ODM 203	I/IIa	Completed	NCT02264418	Advanced or metastatic solid tumors (*n* = 84)	[263]
**TLR2, TLR4**	OM-174	I	Completed	NCT01800812	Solid tumors (*n* = 27)	[264]
**PDGFR**	Sorafenib	IV	Recruiting	NCT02733809	HCC (*n* = 40)	NA
**IGFR**	Figitumumab	I	Completed	NA	Solid tumors (*n* = 24)	[265]
**JAK1/2**	AZD1480 (Antisense oligonucleotide)	I	Terminated	NCT01112397	Solid malignancies (*n* = 72)	NA
		I	Terminated	NCT01219543	HCC, NSCLC (*n* = 47)	NA
	Ruxolitinib (Small molecule)	II	Completed	NCT00674479	AML, ALL, CML (*n* = 51)	[266]
	Momelotinib (Small molecule)	I	Terminated	NCT02258607	NSCLC (*n* = 21)	[267]
		III	Terminated	NCT02101021	PDAC (*n* = 25)	NA
	INCB018424	I/II	Completed	NCT00509899	Myelofibrosis (*n* = 153)	[268]
**JAK2**	WP1066 (Small molecule)	I	Completed	NCT01904123	Brain tumors (*n* = 8)	NA
	TQ05105 (Small molecule)	I	Recruiting	NCT04339400	Myeloproliferative neoplasms (*n* = 50)	NA
	Fedratinib (Small molecule)	I	Completed	NCT01836705	Neoplasm malignant (*n* = 60)	NA
		I	Completed	NCT01437787	Myelofibrosis (*n* = 289)	[269]
		II	Completed	NCT01523171	Myelofibrosis (*n* = 97)	[270]
	SB1518 (Small molecule)	I/II	Completed	NCT00719836	AML, CML, Myelofibrosis (*n* = 76)	[271]
	LY2784544 (Small molecule)	II	Active	NCT01594723	Myeloproliferative neoplasms (*n* = 110)	[272]
	Lestaurtinib	II	Completed	NCT00494585	Leukemia, Myelofibrosis (*N* = 27)	[273]
**SHP1**	SC-43	I	Withdrawn	NCT03443622	Refractory solid tumor	NA

*BC* Breast cancer, *HNSCC* Head and neck squamous cell carcinoma, *NSCLC* Non-small cell lung cancer, *CRC* Colorectal cancer, *GAC* Gastric adenocarcinoma, *HCC* Hepatocellular carcinoma, *NHL* Non-Hodgkin Lymphoma, *CLL* Chronic lymphocytic leukemia, *SLL* Small lymphocytic lymphoma, *SMM* Smoldering Multiple Myeloma, *IMM* Indolent Multiple Myeloma, *RCC* Renal cell cancer, *CML* Chronic myelogenous leukemia, *PDAC* Pancreatic Ductal Adenocarcinoma, *AML* Adult acute myeloid leukemia, *NA* Not available

### Direct inhibitors

The structure-based approach is the main strategy to develop the direct STAT3 inhibitor. The N-terminal domain, coiled-coiled domain, linker domain, especially DNA-binding domain and SH2 domain of STAT3 are endowed with protein-DNA interacting and protein-protein interacting function, which makes STAT3 amenable to direct targeting. These directly inhibitors are generally classified into four categories: small molecule inhibitors, peptide-based inhibitors, antibody-based inhibitors and oligonucleotide-based inhibitors.

The N-terminal domain of STAT3 is involved in the STAT dimers in forming the tetramers and chromatin structure remodeling [274]. And the coiled-coiled domain is responsible for the recruitment of STAT3 to the membrane IL-22 receptors and nuclear translocation. Until now, ST3-H2A2 [181] and MS3–6 [182] have been the only 2 N-terminal and coiled-coiled domain-based inhibitors of STAT3. MS3–6 is an intracellular expression monobody fused to an E3 ubiquitin ligase substrate receptor VHL, which owns high affinity towards STAT3 with an extremely low K_D_ value = 31 ± 6 nM [182].

The primary functions of DBD are binding to interferon-γ activation site (GAS) sequences or human serum-inducible element within the promotor sites of specific target genes, resulting in the transcription. Consequently, blocking the function of DBD is one of the most important strategies for developing STAT3 direct inhibitors. Small molecules such as InS3-54A18, galiellalactone, SG-1709, SG-1721, GPA512, HJC0152, silibinin, HO-3876, LC28, MMPP, bruceantinol, peptide aptamers such as DBD-1-9R, oligonucleotides such as 15-mer duplex ODN, and platinum-based compounds such as CPA-1 and CPA-7 are inhibitors targeting the DBD domain. Bruceantinol, a recently reported STAT3 DBD selective inhibitor, dramatically attenuates the proliferation of colorectal cancer (CRC) cells with a nanomolar concentration. Moreover, 3 pM of bruceantinol is enough to inhibit STAT3 binding to the target genes (IC50 = 2.4 pM). Notably, BBI-608 (Napabucasin), is the most promising STAT3 inhibitor that may successfully pass the clinical trials, which now have entered clinical phase III investigation. BBI-608 is a first-in-class cancer stemness inhibitor. It significantly inhibits the c-Myc, β-catenin, Nanog and Sox2 mediate cancer stemness [275]. Many early phase I and II clinical trials have determined the safety and efficacy of monotherapy and in combination with standard chemotherapies [276]. Another phase III trial assessed the overall survival rate of napabucasin monotherapy in 282 refractory advanced colorectal cancer patients. The overall survival rate was not significantly different. However, in a prespecified biomarker analysis of pSTAT3-positive patients, the napabucasin group displayed a longer survival than the placebo group (NCT01830621).

So far, BPMB is the only one inhibitor selectively targeting the linker domain of STAT3. Tatsuya’s group found that BPMB blocks the STAT3 activation through the acylation of the linker domain. One group specifically examined the effects of an array of mutants in the STAT3 linker domain, and they found that STAT3 linker domain mutants play an essential role in inhibiting STAT3 transcriptional activation because there were functional interactions between the linker and the DNA binding domain and the SH2 domain [277]. These researches reveal the hidden functions of linker domain and provide new strategy for developing STAT3 inhibitors.

The SH2 domain is the most favorable and best-described target domain. It has a dual- mechanistic function, one is being recruited to the receptors for phosphorylating and the other is binding to the phosphor-tyrosine-peptide ligand of another STAT3 monomer then forming the functional STAT3 dimers and promoting the subsequent biological activities. These features provide the druggable site although STAT3 has no enzymatic activity to target. Until now, numerous inhibitors selectively targeting SH2 have been extensively studied; examples include but are not restricted to the following inhibitors (Table 2). We reviewed several representative inhibitors based on the category of small molecules, peptides and oligonucleotides. Small molecules such as proscillaridin A, periplogenin, MM-206, S3I-201, S3I-1757, stattic, STA-21 and its derivatives, N4, and SD-36, the peptides or peptidominetics such as PY*LKTK, ISS610, CJ-1383 and PM-73G, and the oligonucleotides such as T40214 and T40231, which display a great potency in the pre-clinic studies (Table 2). Proscillaridin A shows a greater inhibitory effect (50 nM) on STAT3 activation compared to S31–201 via interacts with SH2 domain of STAT3 [278]. Some small molecules including OPB-51602, OPB-31121 and C188–9 are undergoing clinical trials (Table 3). One example is periplogenin, a STAT3 inhibitor recently found by our group. It is a natural compound derived from *Streptocaulon juventas*, with potent anti-tumor effects in vitro and in vivo [33]. SD-36 is a potent and selective degrader of STAT3 based on an emerging proteolysis targeting chimera (PROTAC) technology. Excitingly, pharmacokinetics and pharmacodynamic analysis demonstrated that SD-36 is well tolerated in immune-competent mice and achieves a long-lasting regression in mice with single i.v. doses of 25 mg/kg, 50 mg/kg and 100 mg/kg [201]. C188–9 (TTI-101) is a small molecule probe, targeting the phosphotyrosine peptide binding site (IC50 = 7.5–20 μΜ, Ki = 37.3 nM) in the SH2 domain [37]. Currently, a dose-escalation phase I study of oral C188–9 is being evaluated in patients with advanced cancer to evaluate the safety, maximum tolerated dose (MTD), pharmacokinetics, and preliminary antitumor activity (NCT03195699). Accordingly, C188–9 is a promising oral drug for clinical application.

The underlying biology and mechanisms of inhibitors are not limited to specific binding to STAT3. New STAT3 selective inhibitors acting via different modes have continually sprung up in this field. For example, STAT3-IN-1 dual-inhibition of the acetylation and phosphorylation of STAT3, displays a potent tumor inhibitory effect in pre-clinical models [217]. Alantolactone, a sesquiterpene lactone component of *Inula helenium*, abrogates STAT3 activation by promoting STAT3 glutathionylation, leading to oxidative stress-dependent apoptosis in lung cancer [279]. Oligonucleotides-based inhibitors break the ‘undruggable’ limit in developing STAT3 inhibitor. AZD9150, the antisense oligonucleotides, which targets the 3′ untranslated region of the STAT3 gene, displays promising efficacy in the pre-clinic models and clinical trials [247]. CTLA4apt-STAT3**,** a CTLA4 aptamer that delivers STAT3 siRNA to tumor cells, CD8^+^ T cells and Treg cells, finally inducing activation of the antitumor immunity [219]. Pyrimethamine, which has been tested in early-phase clinical trials, shows a dual inhibitory of STAT3 activation combined with immune response [280]. Recently, more and more research has switched attention to the area of mtSTAT3. The OPB-51602 and the OPB-111077 (phase I) are mtSTAT3 inhibitors through inhibition of OXPHOS and increase ROS production of mitochondrial ETC and thus induce mitophagy and cell death [281, 282]. MDC-1112, another mtSTAT3 inhibitor, inhibits the mitochondrial accumulation of mtSTAT3 and thus leads to depolarized mitochondrial membrane potential and increased ROS production [283].

### Indirect inhibitors

As mentioned above, the activation of STAT3 signaling is commonly regulated by ligands which interact with cognate membrane receptors and thus activate STAT3; the membrane receptors and associated kinases including cytokine receptors, GPCRs, TLRs, RTKs, non-RTKs, serine/threonine kinases which directly or indirectly activate STAT3. The negative regulators such as SOCS1, SOCS3, PIAS and protein tyrosine phosphatases commonly lead to the disruption of STAT3 activation. Therefore, targeting these regulators has emerged as a prominent strategy for developing the STAT3 indirect inhibitors. The detailed information is provided in Tables 2 and 3.

Siltuximab and tocilizumab, are two FDA-approved monoclonal antibodies targeting IL-6 and IL-6R, separately. The antibody-based inhibitors generally show high affinity and specificity [252]. LY2784544 (Gandotinib), a potent, selective, small-molecule inhibitor of JAK2 and JAK2 V617F, is the most promising JAK2 inhibitor that would enter the phase III clinical trial [272]. Cirsiliol, a novel inhibitor of TYK2 recently reported by our group, interacts with TYK2 with a high affinity (KD = 0.8 μM). Moreover, orally with dose of 10 mg/kg, 50 mg/kg of cirsiliol displayed efficacious tumor inhibitory effect in ESCC PDX models [284]. SC-43, SC-59, SC-78, the potent and orally agonists of SHP-1, significantly augment SHP-1 activity and attenuate the phosphorylation of STAT3 [235, 236]. Moreover, SC-43 has been entered into clinical trial investigation (NCT03443622).

### Combination therapy

#### Targeting STAT3 in combination with chemotherapy or radiotherapy

Compelling evidence demonstrates that STAT3 inhibitors enhance the efficacy of established therapeutic agents in both preclinical and clinical investigations. STAT3 decoy ODNs combined with irradiation and methotrexate show a better efficacy than singe irradiation or methotrexate treatment in metastatic breast cancer cell line [285]. CpG-STAT3ASO, an antisense oligonucleotide combined with radiotherapy could activate the mature of human DCs, M1 polarization of macrophages and promoted CD8^+^ T cell recruitment, thereby suppressing UM-SCC1 tumor growth [286]. Due to the excellent efficacy of BBI-608, several early-phase clinical trials have been performed to test the combination with paclitaxel in patients with platinum-resistant ovarian cancer, melanoma, bladder cancer, NSCLC, gastric adenocarcinomas, pancreatic adenocarcinoma, triple-negative breast cancer. Other phase III trials are also ongoing with napabucasin in combination with the 5-fluorouracil (5-FU), leucovorin, irinotecan (FOLFIRI) in patients with previously treated metastatic colorectal cancer (NCT02753127, NCT03522649).

#### Targeting STAT3 in combination with targeted therapy

Much evidence has shown that blockade of various RTKs contributes to the feedback loop of STAT3 activation, and thus leads to drug resistance and therapeutic failure [171, 175]. Accordingly, simultaneously disrupting the STAT3 signaling, and targeted therapy, would be more promising and effective than monotherapy in human cancers. Wen’s group also has shown that AZD1480 synergy with EGFR inhibitor (gefitinib) decreased human ovarian cancer tumor growth much more robustly than either agent alone in vitro and in vivo [287]. TG101348, a highly selective ATP-competitive JAK2 inhibitor, potentiated the antitumor effect of erlotinib in EGFR-mutant NSCLC [288]. Combination of trastuzumab and S31–201 suppressed the growth of the trastuzumab-resistant HER2-positive breast and gastric cancer tumor xenografts [289]. Consequently, parallel application of the STAT3 inhibitors with FDA-approved RTKs targeted therapy provides a promising strategy for future clinic investigation.

#### Targeting STAT3 in combination with immunotherapy

As discussed above, STAT3 participates in almost all aspects of immune escape and tolerance in TME. Consequently, combination STAT3 inhibition with immune checkpoint inhibitor is a promising strategy to improve the clinical response in tumor patients. STX-0119, a STAT3 dimerization inhibitor, combined with nivolumab (anti-PD-1 antibody), shows more dramatic inhibitory effects on the tumor growth and tumor-infiltrating lymphocyte numbers than STX-0119 and nivolumab single treatment in PANC-1 pancreatic cancer cells xenograft [290]. Recently, one group found that patients with chemotherapy-refractory metastatic PDAC simultaneously treated with MEK inhibitor (Trametinib), STAT3 inhibitor (Ruxolitinib), and PD-1 inhibitor (Nivolumab) not only had good tolerance, but also yielded significant clinical benefit through enhancing the CD4^+^ and CD8^+^ T-cell recruitment and reducing the populations of immunosuppressive TAMs and MDSCs in the PDAC TME [291]. GPB730, another STAT3 inhibitor, combined with anti-CTLA-4 treatment results in significant antitumoral activity and prolonged survival compared to GPB730 or anti-CTLA-4 individual treatment [292]. Huang et al. recently reported that nivolumab in combination with stattic enhances the efficacy and immune response of anti-PD-1 in the immunocompetence for melanoma cells xenograft model through boosting the expression of TIM-3 in CD8^+^ T cells and decreasing the immune-suppressive cytokines IL-10 and TGF-β production in Treg cells [293]. STAT3 direct inhibitor BBI-608 combined with different immunotherapeutic agents such as ipilimumab, pembrolizumab, and pembrolizumab is also indicated for clinical trials [294]. AZD9150 combined with durvalumab (anti-PD-L1), other indirect inhibitors such dasatinib combined with ipilimumab (anti-CTLA-4), dasatinib combined with nivolumab, ruxolitinib combined with pembrolizumab (anti-PD-1), and cetuximab combined with pembrolizumab are ongoing in the phase I/II clinical trials [18]. Due to the promising results of these pre-clinical models and early phase clinic trials, further development of combination STAT3 inhibition with immunotherapy is urgently needed.

Furthermore, cancer vaccines are also a promising strategy for immunotherapy. They work more efficiently to deliver the antigens and easily have uptake by DCs and act with a desired immune response. SVMAV, is one nonvaccine construct with one specific antigen with TLR7/8 agonist to stattic. The combination therapy of SVMAV and anti-PD-1 antibody shows a synergistic inhibitory effect and prolongs the survival duration of melanoma-bearing mice compared with anti-PD-1 single treatment in mice [295]. Similarly, CpG is a TLR9 agonist that triggers TLR9^+^ cells such as DCs, macrophages and B cells induced antitumor immune response. CpG-STAT3 siRNA, is a tumor vaccine that co-delivers CpG and STAT3 siRNA oligonucleotide, achieving a whole-body immune response and inhibiting the immune suppressive environment in TME [296].

Since STAT3 has well-described roles in myeloid cells and Treg in promoting the immunosuppressive effects and attenuating the antitumor effects of CD8^+^ cells. Chimeric antigen receptor T (CAR-T) therapy has been approved by FDA and shows encouraging antitumor effects on human hematologic malignancies. Therefore, STAT3 inhibition combined with CAR-T cell therapy has become a new and emerging therapy. Maciej’s group deletes STAT3 alleles in both CD4^+^ and CD8^+^ T cells prior to implantation, significantly inhibiting the growth of melanoma tumors in mice compared with STAT3^+/+^ CD8^+^ T cells [131]. They also tried to block STAT3 signaling using the inhibitor sunitinib in conjunction with T cells prior to transfer, showing infiltration, expansion and stimulation of T cells came to with similar results. Furthermore, tocilizumab, (STAT3 indirect inhibitor, anti-IL-6 antibody) combines with anti-CD19 CAR-T, one phase I clinical trial to determine efficacy in the CART19 associated cytokine release syndrome (NCT02906371).

#### Targeting STAT3 mediated metabolic reprogramming of cancer cells and immune cells

The strategies for targeting cancer cell metabolism often ignore the metabolism of non-cancerous mesenchymal cells and immune system cells which contribute to the tumor progression [297]. Thus, uncovering the mechanism of metabolic reprogramming of cancer cells and tumor immune cells is a promising area to develop new methods to conquer the immunosuppression in cancer [298]. Recently, STAT3 has been reported to mediate metabolic reprogramming of MDSCs to lipid uptake and FAO in TME. Pharmacological inhibition of FAO attenuates the activity of MDSCs and tumor growth [298]. In addition, the metabolic reprogramming to lipid metabolism and the contribution of OXPHOS to the activation of M2 macrophages [298] is also regulated by STAT3 through another mechanism mentioned in this review. Based on these discussions, combination targeting the Warburg effects, fatty acid oxidation, and amino acid metabolism regulated by STAT3 with targeting the enzymes involved in metabolic reprogramming in immune cells may have therapeutic potential in tumor patients.

## Perspectives and future directions

The unambiguous roles of STAT3 in tumor proliferation, metastasis, angiogenesis, immunosuppression, metabolism reprogramming, drug resistance, CSCs and exosome have been demonstrated by most authoritative assessments. Fascinatingly, despite its importance in embryonic development, STAT3 is dispensable in normal cells and tissues with cumulative evidence [299]. These features make STAT3 a promising target in cancer treatment. Many advances and major endeavors in the field of STAT3 inhibitors from “undruggable” to “druggable” have been made in the past few decades. However, the overall success rate of all drugs entering clinical trials is just 10.4% [300]. More frustrating, no STAT3 targeting drugs have successfully passed the late phase clinical trials. Therefore, the unpredictable complexity of developing STAT3 inhibitors still needs further developments and progress.

The reasons for the STAT3 inhibitors’ not having entered the clinical trials or have failed in the clinical trials were primarily due to the irreversible side effects or lower efficacy. Although the oligonucleotide-based STAT3 inhibitors have high affinity and specificity to STAT3, low cell penetrance and rapid degradation restrict the efficiency of the drug delivery to the tumors. Peptide-based STAT3 inhibitors, also have the common challenges including instability, low delivery efficiency, and the unfavorable pharmacodynamics. Small molecule inhibitors of STAT3 commonly face a low overall rate of entering the clinical practice. The vast majority of small molecules and natural compounds have outstanding inhibitory activities in vitro but lower effects or even contradictory results in vivo. Moreover, poor solubility, bioavailability and high toxicity to normal cells also limit their efficacy in early clinical trials. Furthermore, despite the fact that advances and breakthroughs have been made in cancer immunotherapy, varying response rates among cancers, immune-related side effects and resistance yet to be solved.

Collectively, the poor cell penetrance, instability, lower bioavailability, side effects and bad targeted delivery efficiency are the common obstacles that need to be addressed. Researchers can attempt to design novel delivery systems of for these inhibitors, such as the nanoscale delivery system or co-delivery strategy. For example, the oligonucleotides-based inhibitors with small molecules, oligonucleotides-based inhibitors with peptides-based inhibitors, or small molecules with peptides-based inhibitors coloaded on nanoparticles for targeting the tumor tissues through the enhanced bioavailability, permeability and retention effect may be helpful [301]. For the immunotherapy, more specific biomarkers, CAR-T cells, immune checkpoint molecules or other technologies such as nano-delivery systems and vaccines need to be identified. The development of inhibitors for next-generation immune checkpoint molecules such as LAG-3, TIGIT, B7-H3, V-domain Ig suppressor of T cell activation (VISTA), B and T cell lymphocyte attenuator (BTLA) and adenosine A2a receptor (A2aR) also provide new insights to drug discovery. Furthermore, the combination of low dose STAT3 inhibitors with chemotherapy, targeted therapy or immunotherapy may improve the efficacy and reduce the side effects of STAT3 inhibitors. This strategy has been discussed above. Anyhow, future research should take various approaches and more technologies in consideration to overcome these drawbacks, and new strategies of targeting STAT3 for therapeutic interventions are also being urgent.

Remarkably, on the one hand, the function of mtSTAT3 creates new possibilities for cancer treatment. However, little is known about the transcriptional activity of mtSTAT3 to regulate mitochondrial DNA. Whether mtSTAT3 regulates other immune cells’ differentiation and effector function is not clear yet. Thus, the mechanisms of mtSTAT3 mediation of tumorigenesis and cancer progression need further development. Moreover, based on the previous discussion, we propose that combination therapy based on blocking the STAT3 transcriptional activity with interfering the mtSTAT3 function may be synergistic in killing the cancer cells. On the other hand, targeting the STAT3-mediated alternative metabolic reprogramming of cancer is also a promising field. However, there is still an increasing demand for developing more potent and selective metabolic inhibitors of targeting STAT3. Since STAT3 functions as the tumor hub in the TME consisting of stromal cells, immune cells, EMT cells, tumor cells and lymphatic vascular cells, targeting STAT3-mediated metabolic reprogramming in cancer cells should take metabolic alterations that affect other components in TME into consideration. Consequently, the global landscape of STAT3-mediated metabolisms in tumor microenvironment is expected to overcome these challenges.

### Supplementary Information

**Additional file 1.**

## Data Availability

Not applicable.

## Abbreviations

STAT3: Signal transducer and activator of transcription 3
NTD: N-terminal domain
SH2: Src homology
TAD: Transactivation domain
ETC: Electron transport chain
ROS: Reactive oxygen species
JAB1: Jun activation domain-binding protein 1
EMT: Epithelial-mesenchymal transition
LPS: Lipopolysaccharides
AT1: Angiotensin type-1
S1PR1: Sphingosine-1-phosphate receptor
BCR-ABL: Breakpoint-cluster region and Abelson leukemia proteins
nRTKs: Non-receptor kinase
LncRNAs: Long non-coding RNAs
MiRNAs: MicroRNAs
CircRNAs: Circular RNAs
CRC: Colorectal cancer cell
3′-UTR: 3′-untranslated region
p300/CBP: p300/CREB-binding protein
PTPs: Protein tyrosine phosphatases
HNSCC: Head and neck squamous cell carcinoma
TME: Tumor microenvironment
DC: Dendritic cell
MDSC: Myeloid-derived suppressor cell
TAM: Tumor-associated macrophages cell
TAN: Tumor-associated neutrophils cell
T cell: T lymphocyte
B cell: Bursa-dependent lymphocyte
NK: Natural killer
PD-1: Programmed death-1
CTLA-4: Cytotoxic T-lymphocyte associated protein 4
TIM-3: T cell immunoglobulin and mucin domain-containing-3
CCL5: C-C Motif Chemokine Ligand 5
Treg cell: T regulatory cell
FOXP3: Forkhead box P3
TNM: Tumor, Node, Metastasis
CXCR3: C-X-C Motif Chemokine Receptor 3
CXCL10: C-X-C Motif Chemokine Ligand 10
ICAM-1: Intercellular adhesion molecule-1
MPTP: Mitochondrial permeability transition pore
GRIM-19: Gene associated with retinoid-IFN-induced mortality 19
mtSTAT3: Mitochondrial STAT3
PKM2: Pyruvate kinase M2
FAO: Fatty acid oxidation
CPT1B: Carnitine palmitoyltransferase 1B
FABP4: Fatty acids binding protein 4
SREBP1: Sterol regulatory element-binding transcription factor 1
FABP6: Fatty acids binding protein 6
TCA: Tricarboxylic acid cycle
SLC1A5: Solute Carrier Family 1 Member 5
CSC: Cancer stemness cell
EVs: Extracellular vesicles
DLTs: Dose-limiting toxicities
MTD: Maximum tolerated dose
CAR-T: Chimeric antigen receptor T cell
