# Supplementary material for: Unraveling the complexity of STAT3 in cancer: molecular understanding and drug discovery
Source: J Exp Clin Cancer Res. 2024 Jan 20;43:23. doi: 10.1186/s13046-024-02949-5 (PMC10799433; doi:10.1186/s13046-024-02949-5)
Supplement: Supplementary file 1 — Additional file 1. [file 13046_2024_2949_MOESM1_ESM.doc]

**Supplementary files:**

Table S1. Upstream regulators of STAT3 in cancers.

| **Types** | **Names** | **Mechanisms** | **Manner** | **Ref** |
| --- | --- | --- | --- | --- |
| **Positive regulators of STAT3** | | | | |
| Cytokines | **IL-6, IL-11, CNTF, LIF, OSM** | These cytokines bind to cognate cytokine receptors which lead to activation of receptors–gp130–gp130 complex/JAKs/STAT3 signaling | Indirect | [1-4] |
| Growth factors | **IGF, PDGF, EGF, FGF** | Stimulating cognate growth receptors to phosphorylate JAKs which then phosphorylate STAT3 | Indirect | [5-8] |
| GPCRs | **Angiotensin II receptor, S1PR1** | Phosphorylating STAT3 at tyrosine 705 | Indirect | [9, 10] |
| TLRs | **TLR2, TLR3, TLR4, TLR7, TLR9** | Phosphorylating STAT3 at tyrosine 705 or serine 727 | Indirect | [11-14] |
| RTKs | **EGFR, FGFR, PDGFR, IGFR** | Phosphorylating STAT3 at tyrosine 705 | Direct | [5, 6, 8, 15] |
| Non-RTKs | **JAK1/2, TYK2, SRC, BCR-ABL** | Phosphorylating STAT3 at tyrosine 705 | Direct | [16-18] |
| Serine/threonine kinase | **ERK1/2,** **GSK3α/3β, JNK1/2** | Phosphorylate STAT3 at serine 727 or threonine 714. | Direct | [19-22] |
| LncRNAs | **Lnc-BM** | Lnc-BM interacts with JAK2 to mediate OSM and IL-6 triggered STAT3 activation | Indirect | [23] |
|  | **ZEB1-AS1** | ZEB1-AS1 binds to IL-11 and promotes IL-11/STAT3 activation | Indirect | [24] |
|  | **HOXD-ASI** | Promoting JAK2/STAT3 signaling pathway, and JAK2 blockade turn over the effects | Indirect | [25] |
|  | **MIAT** | MIAT upregulates HMGB1 expression and promotes IL-6/JAK2/STAT3 signaling | Indirect | [26] |
|  | **DANCR** | DANCR directly binds with STAT3 to promote STAT3 activation. | Direct | [27] |
|  | **TNK2-AS1** | TNK2-AS1 interacts with STAT3 to increases its protein stability by protecting it from polyubiquitination and proteasome-mediated degradation | Direct | [28] |
|  | **PVT1** | PVT1 directly interacts with p-STAT3 Tyr705 and increases the protein stability by protecting STAT3 from polyubiquitination and proteasome-mediated degradation | Direct | [29] |
|  | **FLANC** | FLANC prolongs the half-life of p-STAT3 Tyr705, but not STAT3 | Direct | [30] |
|  | **ITIH4-AS1** | ITIH4-AS1 interacts with STAT3 and evokes nuclear translocation of phosphorylated STAT3 | Direct | [31] |
|  | **Lnc-UICC** | Lnc-UICC interacts with the phospho-STAT3, and increases the protein stability by protecting it from proteasome-dependent degradation | Direct | [32] |
| MicroRNAs | **MiR-155-5p, MiR-30d, MiR-155** | These microRNAs activate STAT3 by negatively regulating socs1 | Indirect | [33-35] |
|  | **MiR-194** | MiR-194 activates STAT3 by negatively regulating socs2 | Indirect | [36] |
|  | **MiR-19a,MiR-221-3p, MiR-222-3p,MiR-4308, MiR-203,MiR-30,MiR-4449** | These microRNAs activate STAT3 by negatively regulating socs3 | Indirect | [37-42] |
|  | **MiR-18a, MiR-96-5p, MiR-182-5p** | These microRNAs activate STAT3 by negatively regulating PIAS3 | Indirect | [43, 44] |
| CircRNA | **CircFAT1** | CircFAT1 directly binds to STAT3 and preventing STAT3 from dephosphorylating by SHP1 | Direct | [45] |
|  | **CircAmotl1** | CircAmotl1 interacts with STAT3 by modulating Dnmt3a and miR-17 | Direct | [46] |
|  | **CircHIPK3** | CircHIPK3 modulates STAT3 via MiR124-3p | Indirect | [47] |
|  | **Hsa_circ_0068871** | Hsa_circ_0068871 regulates FGFR3 expression and activates STAT3 by targeting miR-181a-5p | Indirect | [48] |
|  | **Circ-E-Cadherin** | Circ-E-Cadherin interacts with EGFR and triggers STAT3 activation | Indirect | [49] |
| **Negative regulators of STAT3** | | | | |
| SOCS family | **SOCS1, SOCS3** | SOCS1/3 interact with JAKs via kinase inhibitory region domain and promotes the JAKs to be degraded by the proteasome | Indirect | [50, 51] |
| E3 SUMO protein ligase | **PIAS3** | PIAS binds to nuclear STAT3, promotes the sumo-modification of STAT3, and finally blocks the DNA-binding activity of STAT3 | Direct | [52] |
| E3 ubiquitin protein ligase | **PDLIM2** | PDLIM2 binds to nuclear STAT3 and promotes the polyubiquitination and proteasomal degradation of STAT3 | Direct | [53] |
| PTPs | **PTPRD, PTPRT, PTPRK, SHPI, SHP2, PTPN1, PTPN2, PTPN9** | Dephosphorylating STAT3 at Tyrosine 705 | Direct | [54] |
| Dual-specificity phosphatase | **DUSP22** | Dephosphorylating STAT3 at Tyrosine 705 | Direct | [55] |
| MicroRNAs | **MiR-15, MiR-16, MiR-17-5p, MiR-29b, MiR-106a/b, MiR-124, MiR-125a, MiR-148a, MiR-320a, MiR-519a, MiR-544, MiR-874, MiR-1181, MiR-1299, MiR-1301, MiR-9600, Let-7a** | These with STAT3 and negatively regulates STAT3 | Direct | [56-71] |
| MicroRNAs | **MiR-9, MiR-26a** | These microRNAs negatively targeting IL-6 | Indirect | [72, 73] |
| MicroRNAs | **Let-7c, MiR-34a, MiR-218** | These microRNAs negatively targeting IL-6R | Indirect | [71, 74, 75] |
| MicroRNAs | **MiR-340-5p** | Inhibiting the phosphorylation of JAK1 | Indirect | [76] |
| MicroRNAs | **MiR-145, MiR-204, MiR-216, MiR-362-3p, MiR-375** | Inhibiting the phosphorylation of JAK2 | Indirect | [77-81] |
| LncRNAs | **LINC00908, Linc-p21** | These lnRNAs interact with STAT3 and inhibit the phosphorylation of STAT3 | Indirect | [82, 83] |

Interleukin (IL); Ciliary neurotrophic factor (CNTF); Leukemia inhibitory factor (LIF); Oncostatin (OSM); Glycoprotein 130 (gp130); Insulin-like growth factor (IGF); Platelet-derived growth factor (PDGF); Epidermal growth factor (EGF); Fibroblast growth factor (FGF); Sphingosine-1-phosphate receptor (S1PR1); G-protein coupled receptors (GPCRs); Toll-like receptors (TLRs); Epidermal growth factor receptor (EGFR), Fibroblast growth factor receptor (FGFR), Platelet-derived growth factor receptor (PDGFR), Insulin-like growth factor receptor (IGFR); Tyrosine-protein kinase (JAK1/2); Tyrosine kinase 2 (TYK2); Steroid receptor coactivator (SRC), Breakpoint-cluster region and Abelson leukemia proteins (BCR-ABL); Extracellular signal-regulated kinase (ERK1/2); Glycogen synthase kinase 3 alpha/3 beta (GSK3α/3β); c-Jun N-terminal kinase 1/2 (JNK1/2); Long non-coding RNAs (LncRNAs); Long noncoding for brain metastasis (Lnc-BM); lncRNA zinc finger E-Box binding homeobox 1 antisense RNA 1 (ZEB1-AS1); Homeobox D cluster antisense RNA 1 (HOXD-ASI); Myocardial infarction-associated transcript (MIAT); Differentiation antagonizing nonprotein coding RNA (DANCR); Tyrosine kinase non receptor 2 antisense RNA 1 (TNK2-AS1); Plasmacytoma variant translocation 1 (PVT1); Inter-Alpha-Trypsin inhibitor heavy chain 4 antisense RNA 1 (ITIH4-AS1); lncRNA upregulated in cervical cancer (Lnc-UICC); MicroRNAs (miRNAs); Circular RNAs (circRNAs); Suppressor of cytokine signaling (SOCS); Protein inhibitor of activated STAT3 (PIAS); PDZ And LIM Domain 2 (PDLIM2); Protein tyrosine phosphatases (PTPs); PTP receptor-type D (PTPRD); PTP receptor-type T (PTPRT); PTP receptor-type K (PTPRK); Src homology region 2 domain-containing phosphatase 1(SHP1); Src homology region 2 domain-containing phosphatase 2 (SHP2); Protein tyrosine phosphatase non-receptor type 1 (PTPN1); T-cell PTP (TC-PTP)/PTP non-receptor type 2 (PTPN2); MEG2/PTP non-receptor type 9 (PTPN9); Dual specificity phosphatase 22 (DUSP22).

Table S2. Target genes of STAT3 in cancers.

| **Targets of STAT3 acting as tumor supporting functions** | | | | |
| --- | --- | --- | --- | --- |
| **Genes** | **Experimental methods** | **Modes of STAT3 regulation** | **Binding sites** | **Ref** |
| **Anti-apoptosis, proliferation and survival** | | | | |
| **BCL-xL** | EMSA | Positive | −600 to 0 bp | [84] |
| **MCL-1** | EMSA | Positive | −94 to −86 bp | [85] |
| **Survivin** | EMSA, ChIP | Positive | −1174 to −1166 bp, −1095 to −1087 bp | [86] |
| **BcL-2** | ChIP | Positive | −1022 to −1002 bp | [87] |
| **Cyclin D1** | EMSA, ChIP | Positive | −984 bp, −568 bp, −475 bp, −239 bp | [88] |
| **Migration and invasion** | | | | |
| **MMP-1** | Luciferase reporter assay | Positive | −500 to + 32 | [89] |
| **MMP-2** | EMSA, ChIP | Positive | −1,340 to −1,120 | [90] |
| **MMP-7** | ChIP | Positive | −304 to +44 | [91] |
| **MMP-9** | ChIP | Positive | −948 to −919 | [92] |
| **Vimentin** | EMSA, ChIP | Positive | −757 to −749 bp | [93] |
| **E-cadherin** | ChIP | Negative | −520 to +70 | [94] |
| **N-cadherin** | WB | Positive | NA | [94] |
| **Twist** | EMSA, ChIP | Positive | −745 and −451 | [95] |
| **ZEB1** | ChIP | Positive | −310 to −130 bp | [94] |
| **Angiogenesis** | | | | |
| **VEGF** | EMSA, ChIP | Positive | −1,340 to −1,120 | [90] |
| **HIF****-1α** | EMSA, ChIP | Positive | −363 to −355 bp | [96] |
| **bFGF** | ChIP | Positive | −997 to −989 bp | [97] |
| **HGF** | EMSA/ChIP | Positive | −149 bp, −110 bp | [98] |
| **CCL5** | EMSA/ChIP | Positive | −474 to −711 | [99] |
| **Immune suppression and inflammation** | | | | |
| **IL-6** | ChIP | Positive | −73 to −54 bp | [100] |
| **IL-10** | EMSA/ChIP | Positive | −120 to −111 bp | [101] |
| **PD-L1** | ChIP | Positive | NA | [102] |
| **TGFβ** | ChIP | Positive | −3155 to −2515 bp | [103] |
| **COX-Ⅱ** | ChIP | Positive | −134 to −127 bp | [104] |
| **NF-κB** | Transcriptional activity assay | Positive | NA | [105] |
| **Metabolic reprogramming** |  |  |  |  |
| **Hexokinase 2** | ChIP | Positive | −1980 to −1800 bp | [106] |
| **LDHA** | ChIP | Positive | NA | [107] |
| **CPT1B** | ChIP | Positive | NA | [108] |
| **CD36** | ChIP | Positive | 16460 – 16468, 37860 – 37868 | [109] |
| **Cancer stemness** |  |  |  |  |
| **Sox2** | WB | Positive | NA | [110] |
| **Nanog** | ChIP | Positive | NA | [111] |
| **c-myc** | EMSA, ChIP | Positive | +84 to +115 bp | [112] |
| **Chemoresistance** | | | | |
| **Oct-4** | WB | Positive | NA | [113] |
| **ABCC2** | QPCR | Positive | NA | [114] |
| **ABCC6** | QPCR | Positive | NA | [115] |
| **P-gp** | QPCR | Positive | NA | [116] |

B-cell lymphoma-extra large (BCL-XL); Myeloid-cell leukemia 1 (MCL-1); B-cell lymphoma 2 (BCL-2); Matrix metalloproteinases (MMP); Hypoxia-inducible factor 1-alpha; Basic fibroblast growth factor (bFGF); Hepatocyte growth factor (HGF); C-C motif chemokine ligand 5 (CCL5); Programmed death-ligand 1 (PD-L1); Transforming growth factor beta (TGFβ); Cyclooxygenase-Ⅱ (COX-Ⅱ); Nuclear factor-κb (NF-κB); Lactate dehydrogenase A (LDHA); Carnitine palmitoyltransferase 1B (CPT1B); SRY-Box transcription factor 2 (Sox2); Nanog homeobox (Nanog); Octamer-binding transcription factor 4 (OCT-4); ATP binding cassette subfamily C member (ABCC); Not available (NA).

**References**

1. Berishaj M, Gao SP, Ahmed S, Leslie K, Al-Ahmadie H, Gerald WL et al. Stat3 is tyrosine-phosphorylated through the interleukin-6/glycoprotein 130/Janus kinase pathway in breast cancer. Breast Cancer Res. 2007; 9:R32.

2. Johnstone CN, Chand A, Putoczki TL, Ernst M. Emerging roles for IL-11 signaling in cancer development and progression: Focus on breast cancer. Cytokine Growth Factor Rev. 2015; 26:489-498.

3. Li X, Yang Q, Yu H, Wu L, Zhao Y, Zhang C et al. LIF promotes tumorigenesis and metastasis of breast cancer through the AKT-mTOR pathway. Oncotarget. 2014; 5:788-801.

4. West NR, Hegazy AN, Owens BMJ, Bullers SJ, Linggi B, Buonocore S et al. Oncostatin M drives intestinal inflammation and predicts response to tumor necrosis factor-neutralizing therapy in patients with inflammatory bowel disease. Nat Med. 2017; 23:579-589.

5. Zong CS, Chan J, Levy DE, Horvath C, Sadowski HB, Wang LH. Mechanism of STAT3 activation by insulin-like growth factor I receptor. J Biol Chem. 2000; 275:15099-15105.

6. Yan JF, Huang WJ, Zhao JF, Fu HY, Zhang GY, Huang XJ et al. The platelet-derived growth factor receptor/STAT3 signaling pathway regulates the phenotypic transition of corpus cavernosum smooth muscle in rats. PLoS One. 2017; 12:e0172191.

7. Chan KS, Carbajal S, Kiguchi K, Clifford J, Sano S, DiGiovanni J. Epidermal growth factor receptor-mediated activation of Stat3 during multistage skin carcinogenesis. Cancer Res. 2004; 64:2382-2389.

8. Bohrer LR, Chuntova P, Bade LK, Beadnell TC, Leon RP, Brady NJ et al. Activation of the FGFR-STAT3 pathway in breast cancer cells induces a hyaluronan-rich microenvironment that licenses tumor formation. Cancer Res. 2014; 74:374-386.

9. Zheng L, Jia X, Zhang C, Wang D, Cao Z, Wang J et al. Angiotensin II in atrial structural remodeling: the role of Ang II/JAK/STAT3 signaling pathway. American journal of translational research. 2015; 7:1021-1031.

10. Liu Y, Deng J, Wang L, Lee H, Armstrong B, Scuto A et al. S1PR1 is an effective target to block STAT3 signaling in activated B cell-like diffuse large B-cell lymphoma. Blood. 2012; 120:1458-1465.

11. Tye H, Kennedy CL, Najdovska M, McLeod L, McCormack W, Hughes N et al. STAT3-driven upregulation of TLR2 promotes gastric tumorigenesis independent of tumor inflammation. Cancer Cell. 2012; 22:466-478.

12. Liu C, Gao F, Li B, Mitchel RE, Liu X, Lin J et al. TLR4 knockout protects mice from radiation-induced thymic lymphoma by downregulation of IL6 and miR-21. Leukemia. 2011; 25:1516-1519.

13. Ochi A, Graffeo CS, Zambirinis CP, Rehman A, Hackman M, Fallon N et al. Toll-like receptor 7 regulates pancreatic carcinogenesis in mice and humans. The Journal of clinical investigation. 2012; 122:4118-4129.

14. Wang C, Cao S, Yan Y, Ying Q, Jiang T, Xu K et al. TLR9 expression in glioma tissues correlated to glioma progression and the prognosis of GBM patients. BMC Cancer. 2010; 10:415.

15. Harada D, Takigawa N, Kiura K. The Role of STAT3 in Non-Small Cell Lung Cancer. Cancers (Basel). 2014; 6:708-722.

16. Garcia R, Bowman TL, Niu G, Yu H, Minton S, Muro-Cacho CA et al. Constitutive activation of Stat3 by the Src and JAK tyrosine kinases participates in growth regulation of human breast carcinoma cells. Oncogene. 2001; 20:2499-2513.

17. Wan J, Fu AK, Ip FC, Ng HK, Hugon J, Page G et al. Tyk2/STAT3 signaling mediates beta-amyloid-induced neuronal cell death: implications in Alzheimer's disease. J Neurosci. 2010; 30:6873-6881.

18. Coppo P, Flamant S, De Mas V, Jarrier P, Guillier M, Bonnet ML et al. BCR-ABL activates STAT3 via JAK and MEK pathways in human cells. British journal of haematology. 2006; 134:171-179.

19. Gkouveris I, Nikitakis N, Karanikou M, Rassidakis G, Sklavounou A. Erk1/2 activation and modulation of STAT3 signaling in oral cancer. Oncology reports. 2014; 32:2175-2182.

20. Waitkus MS, Chandrasekharan UM, Willard B, Tee TL, Hsieh JK, Przybycin CG et al. Signal integration and gene induction by a functionally distinct STAT3 phosphoform. Molecular and cellular biology. 2014; 34:1800-1811.

21. Nitta RT, Del Vecchio CA, Chu AH, Mitra SS, Godwin AK, Wong AJ. The role of the c-Jun N-terminal kinase 2-alpha-isoform in non-small cell lung carcinoma tumorigenesis. Oncogene. 2011; 30:234-244.

22. Courapied S, Sellier H, de Carne Trecesson S, Vigneron A, Bernard AC, Gamelin E et al. The cdk5 kinase regulates the STAT3 transcription factor to prevent DNA damage upon topoisomerase I inhibition. J Biol Chem. 2010; 285:26765-26778.

23. Wang S, Liang K, Hu Q, Li P, Song J, Yang Y et al. JAK2-binding long noncoding RNA promotes breast cancer brain metastasis. The Journal of clinical investigation. 2017; 127:4498-4515.

24. Wang Q, Du X, Yang M, Xiao S, Cao J, Song J et al. LncRNA ZEB1-AS1 contributes to STAT3 activation by associating with IL-11 in B-lymphoblastic leukemia. Biotechnology letters. 2017; 39:1801-1810.

25. Zheng L, Chen J, Zhou Z, He Z. Knockdown of long non-coding RNA HOXD-AS1 inhibits gastric cancer cell growth via inactivating the JAK2/STAT3 pathway. Tumour biology : the journal of the International Society for Oncodevelopmental Biology and Medicine. 2017; 39:1010428317705335.

26. Zhu X, Liu L, Wang Y, Cong J, Lin Z, Wang Y et al. lncRNA MIAT/HMGB1 Axis Is Involved in Cisplatin Resistance via Regulating IL6-Mediated Activation of the JAK2/STAT3 Pathway in Nasopharyngeal Carcinoma. Front Oncol. 2021; 11:651693.

27. Zhang X, Yang J, Bian Z, Shi D, Cao Z. Long noncoding RNA DANCR promotes nasopharyngeal carcinoma progression by interacting with STAT3, enhancing IL-6/JAK1/STAT3 signaling. Biomedicine & pharmacotherapy = Biomedecine & pharmacotherapie. 2019; 113:108713.

28. Wang Y, Han D, Pan L, Sun J. The positive feedback between lncRNA TNK2-AS1 and STAT3 enhances angiogenesis in non-small cell lung cancer. Biochemical and biophysical research communications. 2018; 507:185-192.

29. Zhao J, Du P, Cui P, Qin Y, Hu C, Wu J et al. LncRNA PVT1 promotes angiogenesis via activating the STAT3/VEGFA axis in gastric cancer. Oncogene. 2018; 37:4094-4109.

30. Pichler M, Rodriguez-Aguayo C, Nam SY, Dragomir MP, Bayraktar R, Anfossi S et al. Therapeutic potential of FLANC, a novel primate-specific long non-coding RNA in colorectal cancer. Gut. 2020; 69:1818-1831.

31. Liang C, Zhao T, Li H, He F, Zhao X, Zhang Y et al. Long Non-coding RNA ITIH4-AS1 Accelerates the Proliferation and Metastasis of Colorectal Cancer by Activating JAK/STAT3 Signaling. Mol Ther Nucleic Acids. 2019; 18:183-193.

32. Su K, Zhao Q, Bian A, Wang C, Cai Y, Zhang Y. A novel positive feedback regulation between long noncoding RNA UICC and IL-6/STAT3 signaling promotes cervical cancer progression. American journal of cancer research. 2018; 8:1176-1189.

33. Baba O, Hasegawa S, Nagai H, Uchida F, Yamatoji M, Kanno NI et al. MicroRNA-155-5p is associated with oral squamous cell carcinoma metastasis and poor prognosis. J Oral Pathol Med. 2016; 45:248-255.

34. Han M, Wang Y, Guo G, Li L, Dou D, Ge X et al. microRNA-30d mediated breast cancer invasion, migration, and EMT by targeting KLF11 and activating STAT3 pathway. J Cell Biochem. 2018; 119:8138-8145.

35. Jiang S, Zhang HW, Lu MH, He XH, Li Y, Gu H et al. MicroRNA-155 functions as an OncomiR in breast cancer by targeting the suppressor of cytokine signaling 1 gene. Cancer Res. 2010; 70:3119-3127.

36. Das R, Gregory PA, Fernandes RC, Denis I, Wang Q, Townley SL et al. MicroRNA-194 Promotes Prostate Cancer Metastasis by Inhibiting SOCS2. Cancer Res. 2017; 77:1021-1034.

37. Collins AS, McCoy CE, Lloyd AT, O'Farrelly C, Stevenson NJ. miR-19a: an effective regulator of SOCS3 and enhancer of JAK-STAT signalling. PLoS One. 2013; 8:e69090.

38. Ye T, Zhong L, Ye X, Liu J, Li L, Yi H. miR-221-3p and miR-222-3p regulate the SOCS3/STAT3 signaling pathway to downregulate the expression of NIS and reduce radiosensitivity in thyroid cancer. Exp Ther Med. 2021; 21:652.

39. Wang X, Li T, Li M, Cao N, Han J. The Functional SOCS3 RS115785973 Variant Regulated by MiR-4308 Promotes Gastric Cancer Development in Chinese Population. Cellular physiology and biochemistry : international journal of experimental cellular physiology, biochemistry, and pharmacology. 2016; 38:1796-1802.

40. Muhammad N, Bhattacharya S, Steele R, Ray RB. Anti-miR-203 suppresses ER-positive breast cancer growth and stemness by targeting SOCS3. Oncotarget. 2016; 7:58595-58605.

41. Che S, Sun T, Wang J, Jiao Y, Wang C, Meng Q et al. miR-30 overexpression promotes glioma stem cells by regulating Jak/STAT3 signaling pathway. Tumour biology : the journal of the International Society for Oncodevelopmental Biology and Medicine. 2015; 36:6805-6811.

42. Yan Z, Hong S, Song Y, Bi M. microR-4449 Promotes Colorectal Cancer Cell Proliferation via Regulation of SOCS3 and Activation of STAT3 Signaling. Cancer Manag Res. 2021; 13:3029-3039.

43. Wu W, Takanashi M, Borjigin N, Ohno SI, Fujita K, Hoshino S et al. MicroRNA-18a modulates STAT3 activity through negative regulation of PIAS3 during gastric adenocarcinogenesis. British journal of cancer. 2013; 108:653-661.

44. Xiao Y, Huang W, Huang H, Wang L, Wang M, Zhang T et al. miR-182-5p and miR-96-5p Target PIAS1 and Mediate the Negative Feedback Regulatory Loop between PIAS1 and STAT3 in Endometrial Cancer. DNA Cell Biol. 2021; 40:618-628.

45. Jia L, Wang Y, Wang CY. circFAT1 Promotes Cancer Stemness and Immune Evasion by Promoting STAT3 Activation. Adv Sci (Weinh). 2021; 8:2003376.

46. Yang ZG, Awan FM, Du WW, Zeng Y, Lyu J, Wu et al. The Circular RNA Interacts with STAT3, Increasing Its Nuclear Translocation and Wound Repair by Modulating Dnmt3a and miR-17 Function. Mol Ther. 2017; 25:2062-2074.

47. Chen X, Mao R, Su W, Yang X, Geng Q, Guo C et al. Circular RNA circHIPK3 modulates autophagy via MIR124-3p-STAT3-PRKAA/AMPKalpha signaling in STK11 mutant lung cancer. Autophagy. 2020; 16:659-671.

48. Mao W, Huang X, Wang L, Zhang Z, Liu M, Li Y et al. Circular RNA hsa_circ_0068871 regulates FGFR3 expression and activates STAT3 by targeting miR-181a-5p to promote bladder cancer progression. Journal of experimental & clinical cancer research : CR. 2019; 38:169.

49. Gao X, Xia X, Li F, Zhang M, Zhou H, Wu X et al. Circular RNA-encoded oncogenic E-cadherin variant promotes glioblastoma tumorigenicity through activation of EGFR-STAT3 signalling. Nat Cell Biol. 2021; 23:278-291.

50. Sharma J, Larkin J, 3rd. Therapeutic Implication of SOCS1 Modulation in the Treatment of Autoimmunity and Cancer. Frontiers in pharmacology. 2019; 10:324.

51. Krebs DL, Hilton DJ. SOCS proteins: negative regulators of cytokine signaling. Stem Cells. 2001; 19:378-387.

52. Chung CD, Liao J, Liu B, Rao X, Jay P, Berta P et al. Specific inhibition of Stat3 signal transduction by PIAS3. Science. 1997; 278:1803-1805.

53. Tanaka T, Yamamoto Y, Muromoto R, Ikeda O, Sekine Y, Grusby MJ et al. PDLIM2 inhibits T helper 17 cell development and granulomatous inflammation through degradation of STAT3. Science signaling. 2011; 4:ra85.

54. Wu M, Song D, Li H, Yang Y, Ma X, Deng S et al. Negative regulators of STAT3 signaling pathway in cancers. Cancer Manag Res. 2019; 11:4957-4969.

55. Sekine Y, Tsuji S, Ikeda O, Sato N, Aoki N, Aoyama K et al. Regulation of STAT3-mediated signaling by LMW-DSP2. Oncogene. 2006; 25:5801-5806.

56. Srinivas C, Ramaiah MJ, Lavanya A, Yerramsetty S, Kavi Kishor PB, Basha SA et al. Novel Etoposide Analogue Modulates Expression of Angiogenesis Associated microRNAs and Regulates Cell Proliferation by Targeting STAT3 in Breast Cancer. PLoS One. 2015; 10:e0142006.

57. Liao XH, Xiang Y, Yu CX, Li JP, Li H, Nie Q et al. STAT3 is required for MiR-17-5p-mediated sensitization to chemotherapy-induced apoptosis in breast cancer cells. Oncotarget. 2017; 8:15763-15774.

58. Qin L, Li R, Zhang J, Li A, Luo R. Special suppressive role of miR-29b in HER2-positive breast cancer cells by targeting Stat3. American journal of translational research. 2015; 7:878-890.

59. Zhang L, Li J, Wang Q, Meng G, Lv X, Zhou H et al. The relationship between microRNAs and the STAT3-related signaling pathway in cancer. Tumour biology : the journal of the International Society for Oncodevelopmental Biology and Medicine. 2017; 39:1010428317719869.

60. Wang S, Wu G, Han Y, Song P, Chen J, Wu Y et al. miR-124 regulates STAT3-mediated cell proliferation, migration and apoptosis in bladder cancer. Oncology letters. 2018; 16:5875-5881.

61. Yang L, Zhang S, Guo K, Huang H, Qi S, Yao J et al. miR-125a restrains cell migration and invasion by targeting STAT3 in gastric cancer cells. OncoTargets and therapy. 2019; 12:205-215.

62. Yu B, Lv X, Su L, Li J, Yu Y, Gu Q et al. MiR-148a Functions as a Tumor Suppressor by Targeting CCK-BR via Inactivating STAT3 and Akt in Human Gastric Cancer. PLoS One. 2016; 11:e0158961.

63. Liang Y, Li S, Tang L. MicroRNA 320, an Anti-Oncogene Target miRNA for Cancer Therapy. Biomedicines. 2021; 9.

64. Hong L, Ya-Wei L, Hai W, Qiang Z, Jun-Jie L, Huang A et al. MiR-519a functions as a tumor suppressor in glioma by targeting the oncogenic STAT3 pathway. J Neurooncol. 2016; 128:35-45.

65. Jin H, Du XJ, Zhao Y, Xia DL. XIST/miR-544 axis induces neuropathic pain by activating STAT3 in a rat model. Journal of cellular physiology. 2018; 233:5847-5855.

66. Zhang X, Tang J, Zhi X, Xie K, Wang W, Li Z et al. miR-874 functions as a tumor suppressor by inhibiting angiogenesis through STAT3/VEGF-A pathway in gastric cancer. Oncotarget. 2015; 6:1605-1617.

67. Wang J, Guo XJ, Ding YM, Jiang JX. miR-1181 inhibits invasion and proliferation via STAT3 in pancreatic cancer. World J Gastroenterol. 2017; 23:1594-1601.

68. Wang Y, Lu Z, Wang N, Zhang M, Zeng X, Zhao W. MicroRNA-1299 is a negative regulator of STAT3 in colon cancer. Oncology reports. 2017; 37:3227-3234.

69. Yang F, Wang H, Yan B, Li T, Min L, Chen E et al. Decreased level of miR-1301 promotes colorectal cancer progression via activation of STAT3 pathway. Biol Chem. 2021; 402:805-813.

70. Sun CC, Li SJ, Zhang F, Zhang YD, Zuo ZY, Xi YY et al. The Novel miR-9600 Suppresses Tumor Progression and Promotes Paclitaxel Sensitivity in Non-small-cell Lung Cancer Through Altering STAT3 Expression. Mol Ther Nucleic Acids. 2016; 5:e387.

71. Patel K, Kollory A, Takashima A, Sarkar S, Faller DV, Ghosh SK. MicroRNA let-7 downregulates STAT3 phosphorylation in pancreatic cancer cells by increasing SOCS3 expression. Cancer letters. 2014; 347:54-64.

72. Zhang J, Jia J, Zhao L, Li X, Xie Q, Chen X et al. Down-regulation of microRNA-9 leads to activation of IL-6/Jak/STAT3 pathway through directly targeting IL-6 in HeLa cell. Mol Carcinog. 2016; 55:732-742.

73. Yang X, Liang L, Zhang XF, Jia HL, Qin Y, Zhu XC et al. MicroRNA-26a suppresses tumor growth and metastasis of human hepatocellular carcinoma by targeting interleukin-6-Stat3 pathway. Hepatology (Baltimore, Md). 2013; 58:158-170.

74. Rokavec M, Oner MG, Li H, Jackstadt R, Jiang L, Lodygin D et al. IL-6R/STAT3/miR-34a feedback loop promotes EMT-mediated colorectal cancer invasion and metastasis. The Journal of clinical investigation. 2014; 124:1853-1867.

75. Yang Y, Ding L, Hu Q, Xia J, Sun J, Wang X et al. MicroRNA-218 functions as a tumor suppressor in lung cancer by targeting IL-6/STAT3 and negatively correlates with poor prognosis. Molecular cancer. 2017; 16:141.

76. Rongxin S, Pengfei L, Li S, Xiaochen J, Yihe H. MicroRNA-340-5p suppresses osteosarcoma development by down-regulating the Wnt/beta-catenin signaling pathway via targeting the STAT3 gene. Eur Rev Med Pharmacol Sci. 2019; 23:982-991.

77. Jiang G, Huang C, Li J, Huang H, Jin H, Zhu J et al. Role of STAT3 and FOXO1 in the Divergent Therapeutic Responses of Non-metastatic and Metastatic Bladder Cancer Cells to miR-145. Molecular cancer therapeutics. 2017; 16:924-935.

78. Li T, Pan H, Li R. The dual regulatory role of miR-204 in cancer. Tumour biology : the journal of the International Society for Oncodevelopmental Biology and Medicine. 2016; 37:11667-11677.

79. Tian YS, Zhong D, Liu QQ, Zhao XL, Sun HX, Jin J et al. Upregulation of miR-216a exerts neuroprotective effects against ischemic injury through negatively regulating JAK2/STAT3-involved apoptosis and inflammatory pathways. J Neurosurg. 2018; 130:977-988.

80. Zhou J, Li Z, Zhao Q, Wu T, Zhao Q, Cao Y. Knockdown of SNHG1 alleviates autophagy and apoptosis by regulating miR-362-3p/Jak2/stat3 pathway in LPS-injured PC12 cells. Neurochem Res. 2021; 46:945-956.

81. Wei R, Yang Q, Han B, Li Y, Yao K, Yang X et al. microRNA-375 inhibits colorectal cancer cells proliferation by downregulating JAK2/STAT3 and MAP3K8/ERK signaling pathways. Oncotarget. 2017; 8:16633-16641.

82. Wang Y, Wu S, Zhu X, Zhang L, Deng J, Li F et al. LncRNA-encoded polypeptide ASRPS inhibits triple-negative breast cancer angiogenesis. J Exp Med. 2020; 217.

83. Jin S, Yang X, Li J, Yang W, Ma H, Zhang Z. p53-targeted lincRNA-p21 acts as a tumor suppressor by inhibiting JAK2/STAT3 signaling pathways in head and neck squamous cell carcinoma. Molecular cancer. 2019; 18:38.

84. Catlett-Falcone R, Landowski TH, Oshiro MM, Turkson J, Levitzki A, Savino R et al. Constitutive activation of Stat3 signaling confers resistance to apoptosis in human U266 myeloma cells. Immunity. 1999; 10:105-115.

85. Epling-Burnette PK, Liu JH, Catlett-Falcone R, Turkson J, Oshiro M, Kothapalli R et al. Inhibition of STAT3 signaling leads to apoptosis of leukemic large granular lymphocytes and decreased Mcl-1 expression. The Journal of clinical investigation. 2001; 107:351-362.

86. Gritsko T, Williams A, Turkson J, Kaneko S, Bowman T, Huang M et al. Persistent activation of stat3 signaling induces survivin gene expression and confers resistance to apoptosis in human breast cancer cells. Clinical cancer research : an official journal of the American Association for Cancer Research. 2006; 12:11-19.

87. Choi HJ, Han JS. Overexpression of phospholipase D enhances Bcl-2 expression by activating STAT3 through independent activation of ERK and p38MAPK in HeLa cells. Biochimica et biophysica acta. 2012; 1823:1082-1091.

88. Leslie K, Lang C, Devgan G, Azare J, Berishaj M, Gerald W et al. Cyclin D1 is transcriptionally regulated by and required for transformation by activated signal transducer and activator of transcription 3. Cancer Res. 2006; 66:2544-2552.

89. Itoh M, Murata T, Suzuki T, Shindoh M, Nakajima K, Imai K et al. Requirement of STAT3 activation for maximal collagenase-1 (MMP-1) induction by epidermal growth factor and malignant characteristics in T24 bladder cancer cells. Oncogene. 2006; 25:1195-1204.

90. Huang W, Yu LF, Zhong J, Wu W, Zhu JY, Jiang FX et al. Stat3 is involved in angiotensin II-induced expression of MMP2 in gastric cancer cells. Dig Dis Sci. 2009; 54:2056-2062.

91. Yuan G, Qian L, Shi M, Lu F, Li D, Hu M et al. HER2-dependent MMP-7 expression is mediated by activated STAT3. Cell Signal. 2008; 20:1284-1291.

92. Song Y, Qian L, Song S, Chen L, Zhang Y, Yuan G et al. Fra-1 and Stat3 synergistically regulate activation of human MMP-9 gene. Mol Immunol. 2008; 45:137-143.

93. Wu Y, Diab I, Zhang X, Izmailova ES, Zehner ZE. Stat3 enhances vimentin gene expression by binding to the antisilencer element and interacting with the repressor protein, ZBP-89. Oncogene. 2004; 23:168-178.

94. Xiong H, Hong J, Du W, Lin YW, Ren LL, Wang YC et al. Roles of STAT3 and ZEB1 proteins in E-cadherin down-regulation and human colorectal cancer epithelial-mesenchymal transition. J Biol Chem. 2012; 287:5819-5832.

95. Cheng GZ, Zhang WZ, Sun M, Wang Q, Coppola D, Mansour M et al. Twist is transcriptionally induced by activation of STAT3 and mediates STAT3 oncogenic function. J Biol Chem. 2008; 283:14665-14673.

96. Niu G, Briggs J, Deng J, Ma Y, Lee H, Kortylewski M et al. Signal transducer and activator of transcription 3 is required for hypoxia-inducible factor-1alpha RNA expression in both tumor cells and tumor-associated myeloid cells. Molecular cancer research : MCR. 2008; 6:1099-1105.

97. Huang YH, Wu MP, Pan SC, Su WC, Chen YW, Wu LW. STAT1 activation by venous malformations mutant Tie2-R849W antagonizes VEGF-A-mediated angiogenic response partly via reduced bFGF production. Angiogenesis. 2013; 16:207-222.

98. Nakagawa K, Takasawa S, Nata K, Yamauchi A, Itaya-Hironaka A, Ota H et al. Prevention of Reg I-induced beta-cell apoptosis by IL-6/dexamethasone through activation of HGF gene regulation. Biochimica et biophysica acta. 2013; 1833:2988-2995.

99. Lee E, Fertig EJ, Jin K, Sukumar S, Pandey NB, Popel AS. Breast cancer cells condition lymphatic endothelial cells within pre-metastatic niches to promote metastasis. Nat Commun. 2014; 5:4715.

100. Wang T, Niu G, Kortylewski M, Burdelya L, Shain K, Zhang S et al. Regulation of the innate and adaptive immune responses by Stat-3 signaling in tumor cells. Nat Med. 2004; 10:48-54.

101. Schaefer A, Unterberger C, Frankenberger M, Lohrum M, Staples KJ, Werner T et al. Mechanism of interferon-gamma mediated down-regulation of interleukin-10 gene expression. Mol Immunol. 2009; 46:1351-1359.

102. Song TL, Nairismagi ML, Laurensia Y, Lim JQ, Tan J, Li ZM et al. Oncogenic activation of the STAT3 pathway drives PD-L1 expression in natural killer/T-cell lymphoma. Blood. 2018; 132:1146-1158.

103. Kinjyo I, Inoue H, Hamano S, Fukuyama S, Yoshimura T, Koga K et al. Loss of SOCS3 in T helper cells resulted in reduced immune responses and hyperproduction of interleukin 10 and transforming growth factor-beta 1. J Exp Med. 2006; 203:1021-1031.

104. Lo HW, Cao X, Zhu H, Ali-Osman F. Cyclooxygenase-2 is a novel transcriptional target of the nuclear EGFR-STAT3 and EGFRvIII-STAT3 signaling axes. Molecular cancer research : MCR. 2010; 8:232-245.

105. Vyas D, Lopez-Hisijos N, Shah P, Deshpande KS, Basson MD, Vyas A et al. A Second-Generation Proteasome Inhibitor and Doxorubicin Modulates IL-6, pSTAT-3 and NF-kB Activity in MDA-MB-231 Breast Cancer Cells. Journal of nanoscience and nanotechnology. 2017; 17:175-185.

106. Ou B, Sun H, Zhao J, Xu Z, Liu Y, Feng H et al. Polo-like kinase 3 inhibits glucose metabolism in colorectal cancer by targeting HSP90/STAT3/HK2 signaling. Journal of experimental & clinical cancer research : CR. 2019; 38:426.

107. Cheng H, Hao Y, Gao Y, He Y, Luo C, Sun W et al. PLCepsilon promotes urinary bladder cancer cells proliferation through STAT3/LDHA pathwaymediated glycolysis. Oncology reports. 2019; 41:2844-2854.

108. Wang T, Fahrmann JF, Lee H, Li YJ, Tripathi SC, Yue C et al. JAK/STAT3-Regulated Fatty Acid beta-Oxidation Is Critical for Breast Cancer Stem Cell Self-Renewal and Chemoresistance. Cell metabolism. 2018; 27:136-150 e135.

109. Gyamfi J, Yeo JH, Kwon D, Min BS, Cha YJ, Koo JS et al. Interaction between CD36 and FABP4 modulates adipocyte-induced fatty acid import and metabolism in breast cancer. NPJ Breast Cancer. 2021; 7:129.

110. Yang J, Liao D, Chen C, Liu Y, Chuang TH, Xiang R et al. Tumor-associated macrophages regulate murine breast cancer stem cells through a novel paracrine EGFR/Stat3/Sox-2 signaling pathway. Stem Cells. 2013; 31:248-258.

111. Lee TK, Castilho A, Cheung VC, Tang KH, Ma S, Ng IO. CD24(+) liver tumor-initiating cells drive self-renewal and tumor initiation through STAT3-mediated NANOG regulation. Cell stem cell. 2011; 9:50-63.

112. Kiuchi N, Nakajima K, Ichiba M, Fukada T, Narimatsu M, Mizuno K et al. STAT3 is required for the gp130-mediated full activation of the c-myc gene. J Exp Med. 1999; 189:63-73.

113. Cheng CC, Shi LH, Wang XJ, Wang SX, Wan XQ, Liu SR et al. Stat3/Oct-4/c-Myc signal circuit for regulating stemness-mediated doxorubicin resistance of triple-negative breast cancer cells and inhibitory effects of WP1066. International journal of oncology. 2018; 53:339-348.

114. Chua PJ, Lim JP, Guo TT, Khanna P, Hu Q, Bay BH et al. Y-box binding protein-1 and STAT3 independently regulate ATP-binding cassette transporters in the chemoresistance of gastric cancer cells. International journal of oncology. 2018; 53:2579-2589.

115. Soleymani Abyaneh H, Gupta N, Radziwon-Balicka A, Jurasz P, Seubert J, Lai R et al. STAT3 but Not HIF-1alpha Is Important in Mediating Hypoxia-Induced Chemoresistance in MDA-MB-231, a Triple Negative Breast Cancer Cell Line. Cancers (Basel). 2017; 9.

116. Zhao L, Bin S, He HL, Yang JM, Pu YC, Gao CH et al. Sodium butyrate increases P-gp expression in lung cancer by upregulation of STAT3 and mRNA stabilization of ABCB1. Anticancer Drugs. 2018; 29:227-233.
